# IFI16 is essential to linking DNA damage and ferroptosis in acute kidney injury

**DOI:** 10.1038/s41419-026-08604-5

**Published:** 2026-03-23

**Authors:** Zhe Qiao, Di Zhou, Tianxing Zhang, Hongshen Lu, Tongxin Ren, Meng Jia, Zhuhan He, Yongqi Han, Cuicui Lu, Jichao Wu, Min Liu, Yu Sun, Ziying Wang, Yi Lu, Wei Tang, Fan Yi

**Affiliations:** 1 The Key Laboratory of Infection and Immunity of Shandong Province, Department of Pharmacology, School of Basic Medical Sciences, Shandong University, Jinan, China; 2 Department of Pharmacy, Shandong Provincial Hospital Affiliated to Shandong First Medical University, Jinan, China; 3 Department of Biochemistry and Molecular Biology, School of Basic Medical Sciences, Shandong University, Jinan, China; 4 Department of Pathogenic Biology, School of Basic Medical Sciences, Shandong University, Jinan, China; 5 National Key Laboratory for Innovation and Transformation of Luobing Theory, Key Laboratory of Cardiovascular Remodeling and Function Research, Chinese Ministry of Education and Chinese Ministry of Health, Qilu Hospital, Shandong University, Jinan, China

**Keywords:** Acute kidney injury, Mechanisms of disease, DNA damage response

## Abstract

Emerging evidence demonstrates the important role of ferroptosis, a novel regulated cell death, in the initiation and progression of acute kidney injury (AKI). However, the activation mechanism of ferroptosis in AKI has not been fully revealed. The pivotal function of interferon inducible protein 16 (IFI16) in DNA damage response (DDR) as DNA sensor and regulator of cell death pathways encouraged us to examine its role in ferroptosis of renal tubular epithelial cells (TECs) in AKI. Here we report that the levels of IFI16 and its mouse ortholog p204 were elevated in the kidney of patients with acute tubular necrosis (ATN) and in TECs of mice with renal ischemia/reperfusion (I/R)-induced AKI (I/R-AKI). Under I/R conditions, tubule-specific p204 deficiency in mice and IFI16 knockout in HK-2 cells significantly ameliorated TEC ferroptosis. Mechanistically, IFI16 binds to poly(ADP-ribose) polymerase 1 (PARP-1) and enhances protein Poly ADP-ribosylation (PARylation), which in turn potentiates the ataxia-telangiectasia mutated (ATM)-p53 signaling contributing to lipid peroxidation and ferrous ion accumulation in TECs. In addition, IFI16-amplified DDR was dependent on its HIN and PYRIN domains. Thus, our findings provide a better understanding of a critical pathogenic axis linking DNA damage to ferroptosis and suggest that targeting IFI16 may be an innovative therapeutic strategy for treating patients with AKI.

## Introduction

Acute kidney injury (AKI) is a critical condition characterized by a sudden decrease in kidney function that occurs within a duration of 7 days [1]. AKI affects approximately 15% of hospitalized patients worldwide and is independently associated with increased mortality [2, 3]. Numerous studies have proven that AKI increases the potential risks of chronic kidney disease (CKD), cardiovascular disease, and end-stage renal disease (ESRD) [4, 5]. As no specific therapies have been developed to prevent, attenuate or treat AKI, the current treatment of AKI relies only on supportive management, such as fluid therapy, diuretics, and dialysis [6].

Risk factors for AKI typically center around ischemia/reperfusion (I/R), nephrotoxicity, and sepsis. Ischemia/reperfusion injury (IRI) in proximal tubular epithelial cells (TECs) is considered the main cause of AKI [7]. Renal I/R results in sustained inadequate oxygen delivery, the accumulation of metabolites, and ATP depletion, which causes progressive injury and death of TECs and inflammatory processes, leading to kidney dysfunction [8]. Recent studies have emphasized the fundamental role of ferroptosis in the pathogenesis of AKI, as it induces an initial wave of renal tubule cell death and an inflammatory response [9, 10]. Hence, therapeutic strategies targeting ferroptosis could be beneficial for early prevention in AKI. The available evidence implies the involvement of DNA damage and DNA damage response (DDR) in the pathogenesis of AKI [11–14]. DDR governs the outcome of AKI in a bimodal manner. Moderate activation of DDR protects against AKI, while hyperactivation of DDR induces cell death and worsens AKI [15, 16]. Recently, several studies have identified intricate and complicated interplay between ferroptosis, ionizing radiation, ATM (ataxia–telangiectasia mutated)/ATR (ATM and Rad3-related), and tumor suppressor p53, suggesting that DNA damage may play a initiating role in the activation of ferroptosis pathway in AKI [17–20]. However, the regulatory mechanisms linking DNA damage and ferroptosis still need to be explored.

Interferon inducible protein 16 (IFI16) and its mouse ortholog p204 are members of the PYRIN and HIN domain-containing protein family. As an intracellular sensor recognizing both self or non-self DNA, IFI16 plays a crucial role in the innate immune system, inflammation, and the regulation of cell death pathways [21–23]. IFI16 is also a known DNA sensor involved in DDR [21, 24]. For instance, in response to etoposide-induced DNA damage, IFI16 binds to p53 and translocation into the cytoplasm, hence inducing a non-canonical mode of STING activation [25]. In addition, IFI16 acts as a BRCA1-associated protein contributing to p53-mediated transmission of DNA damage signals and apoptosis [26]. Therefore, IFI16 stands as a sentinel at the intersection of DDR, inflammation, and cell fate decisions. However, the precise role of IFI16/p204 in AKI remains to be elucidated.

Here, we show that IFI16/p204 is highly induced in human and mouse TECs under I/R conditions in vitro and in vivo. Using tubule-specific p204-deficient mice and IFI16 knockout HK-2 cells, we demonstrate that IFI16/p204 contributes to ferroptosis of TECs in renal ischemia/reperfusion-induced AKI (I/R-AKI) by amplifying the activation of DDR pathways. This study identifies IFI16/p204 act as a key regulator in cell fate decisions of TECs and an attractive therapeutic target for AKI aimed at inhibiting ferroptosis.

## Materials and methods

### Human renal biopsy samples

Renal biopsies were conducted as part of routine clinical diagnostic investigations, and the samples were procured from the Department of Pathology at Shandong University School of Basic Medical Sciences. Human renal biopsy samples were collected from patients diagnosed with biopsy-proven acute tubular necrosis (ATN), as detailed in Table S1. None of these patients had initiated dialysis therapy at the time of kidney biopsy. Normal control samples were obtained from the healthy kidney poles of individuals who underwent tumor nephrectomies or renal cystectomy, with no other kidney diseases present. All investigations were carried out in accordance with the principles of the Declaration of Helsinki and were approved by the Research Ethics Committee of Shandong University following the acquisition of informed consent from the patients.

### Animal studies

All animal study protocols were approved by the Institutional Animal Care and Use Committee of the School of Basic Medical Sciences, Shandong University (document no. ECSBMSSDU2019-2-024) and conducted in accordance with the National Institutes of Health Guide for the Care and Use of Laboratory Animals. Group allocation was performed in a randomized manner, and investigators were blinded to the allocation of different groups during surgery and outcome evaluations. Mice (five per cage) were housed under standard laboratory conditions, with a 12-h light-dark cycle (lights on at 7:00 a.m.) and a temperature of 24 °C, with ad libitum access to autoclaved water and standard laboratory chow diet (Beijing Keao Xieli Feed, Beijing, China). Cages with standard corncob bedding were changed three times a week. For all of the in vivo experiments, littermate control mice were used. The mouse genotype did not cause visible changes in initial weight, health, or immune status. In all of the experiments, considering that female mice are resistant to renal ischemia reperfusion injury, only male mice were used. Power Analysis and Sample Size (PASS15) software was employed to process the sample size calculation of one-way analysis of variance studies. The power (1-β) was set to 0.9, the significance level α was set to 0.05, and the number of groups was set to 4 with equal allocation ratio. The number of mice used for the experiments is indicated for each experiment in the figure legends. All of our experimental animals were kept under barrier conditions under constant veterinary supervision and did not display any signs of distress or pathological changes that warranted veterinary intervention. Animals demonstrating sickness were euthanized and excluded. Otherwise, all data were included in the study.

### Generation of tubule-specific p204 knockout mice

Floxed p204 mice (B6;129-Ifi204tm1Nju, stock no. B008569, Gempharmatech, China) were hybridized with transgenic mice expressing Cre-recombinase under the cadherin 16 promoter (B6.Cg-Tg(Cdh16-Cre) 91Igr/J, stock no. 012237, Jackson Laboratory, Bar Harbor, ME) to generate tubule-specific p204 knockout mice (Cdh16-Cre^+^/p204^fl/fl^; Cre^+^/p204^fl/fl^). Age-matched mice without Cre (Cdh16-Cre^−^/p204^fl/fl^; Cre^−^/p204^fl/fl^) were used as controls. Genomic DNA isolated from mouse tails was subjected to PCR-based genotyping. The specific primer sets employed for tail PCR genotyping in this study are detailed in Table S2.

### AKI mouse model

I/R induced AKI mouse model was established according to previously described protocols. In brief, 8-week-old male mice (average weight: 20 ± 2 g) were anesthetized with intraperitoneal pentobarbital sodium (50 mg/kg body weight) and subjected to a midline abdominal incision. Bilateral renal pedicles were occluded for 30 min using microaneurysm clamps. Following the ischemic period, the clamp was removed, and all animals were intraperitoneally administered 0.5 ml of warm saline before abdominal closure. Sham operations were performed to exposure of the bilateral kidneys for 30 min, but without the induction of ischemia. The animals were allowed to recover with free access to food and water. Throughout the ischemic period, body temperature was maintained between 36 and 37.5 °C using a temperature-controlled heating system. At the indicated time points after reperfusion, blood samples were collected via cardiac puncture, and anesthetized mice were perfused with phosphate-buffered saline (PBS) at 150–160 mm Hg via a butterfly 23G needle inserted into the left ventricle to completely remove blood. One kidney was then harvested, and the other kidney was further perfused with 4% paraformaldehyde for histopathological analysis. Serum creatinine (SCr) and blood urea nitrogen (BUN) were measured by Kingmed Diagnostics (China) to evaluate the renal function. To determine the expression of p204 in the IRI model, 8-week-old male wild-type C57BL/6 N mice (*n* = 30), purchased from Beijing Vital River Laboratory Animal Technology, were randomly allocated into five groups (*n* = 6/group) as follows: (1) Sham-operated group, (2) I/R 6 h group, (3) I/R 12 h group, (4) I/R 24 h group, and (5) 48 h group. To determine the role of tubule-specific p204 deficiency in the IRI model, 8-week-old male Cre^−^/p204^fl/fl^ mice (*n* = 16) and Cre^+^/p204^fl/fl^ mice (*n* = 16) were randomly allocated into four groups (*n* = 8/group) as follows: (1) Sham-operated Cre^−^/p204^fl/fl^ group, (2) Sham-operated Cre^+^/p2040^fl/fl^ group, (3) I/R 24 h Cre^−^/p204^fl/fl^ group, (4) I/R 24 h Cre^+^/p204^fl/fl^ group, (5) I/R 48 h Cre^−^/p204^fl/fl^ group, and (6) I/R 48 h Cre^+^/p204^fl/fl^ group.

### Histological analysis

Kidney tissues were fixed in 4% paraformaldehyde (PFA), embedded in paraffin, and then sectioned at 4-μm thickness. The sections were stained with hematoxylin and eosin (H&E) and analyzed using an Olympus BX53 (Olympus, Tokyo, Japan) microscope. The extent of tubular injury was assessed by quantifying the percentage of tubules exhibiting cellular necrosis and loss of brush border. This evaluation was performed in a blinded manner using a scoring system as follows: 0, no damage; 1, 0%–10% damage; 2, 11%–25% damage; 3, 26%–45% damage; 4, 46%–75% damage; and 5, >75% damage. A minimum of 8 high-power fields per section for each sample were examined to ensure accurate scoring.

### Immunofluorescence and immunohistochemistry

Paraffin-embedded renal sections were deparaffinized in xylene, rehydrated in graded alcohol, and antigen retrieval using EDTA/EGTA buffer (pH 9.0) at 100 °C for 20 min. Endogenous peroxidase was blocked with 0.3% H_2_O_2_ for 15 min for immunohistochemistry (IHC). Following washing in PBST buffer, the sections were blocked with 5% bovine serum albumin (BSA) for 1 h. Subsequently, primary antibodies were added and incubated at 4 °C overnight, and then visualized using HRP-conjugated secondary antibodies for IHC or fluorescently labeled secondary antibodies for immunofluorescence (IF). For IHC, after secondary antibody incubation and washing, the sections were placed in the solution for the DAB reaction and examined with a microscope (2–10 min). For IF, nuclei were stained with DAPI. The staining was carefully quantified in each slide for each kidney in 8 randomly chosen fields in a blinded manner, and the data were analyzed using Image J 1.45 software (National Institutes of Health, Bethesda, USA). The primary antibodies used were indicated in Table S3.

The IHC of GPX4, SLC7A11, and FTL was scored by a semi-quantitative method using the percentage and intensity of positively stained cells. Positive staining was scored as follows: “0” (less than 5% positively stained cells), “1” (5–24% positively stained cells), “2” (25–49% positively stained cells), “3” (50–74% positively stained cells), and “4” (75–100% positively stained cells). The intensity was scored as follows: “0” (negative staining); “1” (weak staining); “2” (moderate staining); and “3” (strong staining). The final IHC score was generated by multiplying the percentage score by the staining intensity score.

### Terminal deoxynucleotidyl transferase dUTP nick-end labeling (TUNEL) assay

Cell death in kidney tissues was examined using an in situ cell death detection kit (KeyGEN BioTECH, KGA1405) according to the manufacturer’s instructions. Frozen kidney sections were fixed in 4% paraformaldehyde (PFA), followed by incubation with proteinase K (20 μg/mL) at room temperature for 10 minutes. Subsequently, the sections were incubated with a TUNEL reaction mixture at 37 °C for 1 h in a humidified, dark chamber. The fluorescence images were captured under an Olympus BX53 (Olympus, Tokyo, Japan) microscope.

### Cell culture and treatment

Immortalized human proximal tubule epithelial cells (HK-2) (American Type Culture Collection, Manassas, VA) were cultured according to previously established protocols. All the cell lines were authenticated by short tandem repeat analysis and were tested for mycoplasma contamination. In vitro hypoxia/reoxygenation (H/R) was induced by subjecting HK-2 cells to a hypoxic environment (0.1% O_2_) for 2 h, followed by reoxygenation at various time points. For experiments detecting early events such as DDR, samples were collected 2 h after reoxygenation. For the detection of later events such as indicators for ferroptosis, apoptosis, necroptosis, and downstream protein expression, samples were collected 24 h after reoxygenation.

### GST pull-down assay

The recombinant GST fusion wild-type IFI16 and its mutant proteins were produced by Abiotech (Jinan, China) using the E. coli BL21 (DE3) expression system. IFI16 mutants include IFI16-ΔPYRIN (NM_005531(del1-87aa)), IFI16-ΔHINA(NM_005531(del191-393aa)), IFI16-ΔHINB(NM_005531(del509-729aa)), IFI16-ΔHINA&B (NM_005531(del191-393aa/del509-729aa)). Anti-GST magnetic beads (MedChemExpress, HY-K0222) were used for pull-down assay. Beads were prepared according to the manual instructions. 500 µL GST-IFI16 and GST-IFI16-mutant mixture were added to 25 µL anti-GST magnetic beads and the mixture was incubated for overnight at 4 °C with gentle rotation. The beads bound with wild-type IFI16 and different mutants were washed with TBST (50 mM Tris-HCl, pH 7.4, 150 mM NaCl, 0.5% Tween-20) three times and collected using magnetic stand. Following, GST-tagged IFI16- or its mutant on beads were detected by coomassie brilliant blue staining and Western blot using an antibody against GST-tag. Then, 1 µg recombinant His-PARP1 protein (MedChemExpress, HY-P74652) was added to GST-tagged IFI16- or its mutant-labeled beads, and GST-labeled beads were used as control. The pull-down incubation was in TBS (50 mM Tris-HCl, pH 7.4, 150 mM NaCl) for 4 h at 4 °C with gently rotating. Then, beads were washed with washing buffer (TBS containing 0.5% Tween and 0.5% Triton X-100) for three times. Protein bound to the beads was eluted by adding 30 μL of 1 × SDS-PAGE loading buffer to each tube and boiling for 10 min at 100 °C. Supernatant containing both bait and target proteins was collected and PARP1 binding to GST-tagged proteins were assayed by Western blot. Uncropped western blot images are displayed in the section “Supplementary Material”.

### Generation of stable cell lines

The CRISPR/Cas9 genome editing approach was used to generate an IFI16 knockout cell line (sgKO-IFI16). HK-2 cells were transduced with a lentivirus containing the plasmid pLenti-Cas9-GV392-sgRNA-IFI16, which contains both CON250 (5′-CGCTTCCGCGGCCCGTTCAA-3′) and a guide sequence against the IFI16 gene (5′-TATACCAACGCTTGAAGACC-3′). Forty-eight hours after transduction, 2–10 µg/mL puromycin (Solarbio, P8230, China) was added to the well based on the antibiotic resistance genes on the vectors. Then, the cells were incubated for 7 days with puromycin for stable cell line selection, and the transfection efficiency was assessed via western blot analysis.

To construct IFI16 mutants bearing different domain deletions, sgKO-IFI16 HK-2 cells were transduced with lentivirus coding wild-type IFI16, IFI16-ΔPYRIN mutant, or IFI16-ΔHINA&B mutant. To prevent Cas9-mediated cleavage of the transduced IFI16 elements in sgKO-IFI16 HK-2 cells, synonymous mutations were introduced into the LV-IFI16 and LV-IFI16 constructs. IFI16 (NM_005531(samesense mut)), IFI16-ΔPYRIN (NM_005531(del1-87aa)) and IFI16-ΔHINA&B (NM_005531(del191-393aa/del509-729aa/samesense mut)) lentiviruses were constructed by Genechem Co. Ltd (Shanghai, China). SgKO-IFI16 HK-2 cells were infected with different lentiviral vectors at a multiplicity of infection (MOI) of 50. After 48 h of incubation, stable cell lines were obtained by adding antibiotic screening.

IFI16 overexpression HK-2 cells were constructed by infection with LV-IFI16-GFP lentiviral vectors (Genechem, Shanghai, China) at a MOI of 50. After 48 h of incubation, stable cell lines were obtained by adding antibiotic screening. For inhibitor treatment, LV-IFI16-GFP HK-2 cells were pre-incubated with PJ34 (50 μM, MedChemExpress, HY-13688A), Ku55933 (10 μM, MedChemExpress, HY-12016), Z-VAD-FMK (20 μM, MedChemExpress, HY-16658B), Nec-1S (10 μM, MedChemExpress, HY-14622A), Ferrostatin-1 (10 μM, MedChemExpress, HY-100579) for 6 h. Subsequently, the cells were subjected to hypoxia with continuous inhibitor supplementation, followed by reoxygenation for 2 or 24 h.

### RNA extraction and real-time RT-PCR

Total RNA was extracted from the frozen renal cortices of mice or cells using TRIzol reagent (Invitrogen, Carlsbad, CA). cDNA was then synthesized using PrimeScript RT Reagent Kit (Takara, Japan). Real-time quantitative RT-PCR (qRT-PCR) was performed using the UltraSYBR Mixture (CWBIO, Beijing, China). The mRNA levels of target genes were analyzed using the Bio-Rad iCycler system (Bio-Rad, Hercules, CA, USA) in conjunction with Bio-Rad CFX Manager 2.1 software (Bio-Rad, California, USA). The relative gene expression was quantified by the 2^−ΔΔCT^ method, and the housekeeping gene β-actin was used as a control for normalization. The primers used in our studies are indicated in Table S2.

### Western blot analyses

The total protein of the renal cortices from mice or HK-2 cells was extracted with RIPA buffer containing a 1% protease inhibitor cocktail, phosphatase inhibitor, and 1% PMSF. Equal amounts of protein were separated by SDS/ PAGE gels, transferred to PVDF membranes (Millipore), and hybridized to an appropriate primary antibody and HRP-conjugated secondary antibody for subsequent detection by ECL chemiluminescence system. The intensity of bands was analyzed by Image J 1.45 software (National Institutes of Health, Bethesda, USA). The primary antibodies used were indicated in Table S3. Uncropped western blot images are displayed in the section “Supplemental Material”.

### Flow cytometry

Cell apoptosis was determined by propidium iodide-annexin V staining as described. Apoptosis was evaluated in cells via dual staining with allophycocyanin (APC)-conjugated Annexin V and 7-AAD. Following harvesting, cells were resuspended in 200 μL of Annexin V binding buffer, which was supplemented with 5 μL of 7-AAD and 5 μL of Annexin V-APC, and then incubated for 15 min at room temperature in the dark. After washing, cells were resuspended in phosphate-buffered saline. Subsequent flow cytometry acquisition was performed on a Cytoflex S instrument, and the resulting data were analyzed using CytExpert software (both from Beckman Coulter).

### BODIPY staining and confocal imaging

Following treatment, HK-2 cells were washed 3 times with PBS, then incubated with a live cell assay reagent BODIPY 581/591 C11 (C10445, Invitrogen, USA) for 30 min at working concentration of 10 μM in the 37 °C. Remove media and wash three times with PBS, and image cells within 2 h of staining. Excitation and emission wavelengths for the confocal microscope pictures were 510/590 nm using a Carl Zeiss LSM 980 laser scanning confocal microscope (Tokyo, Japan).

### GSH Assay

The GSH content in renal tissues and cells were detected with a GSH/GSSG assay kit (S0053, Beyotime, Shanghai, China). Homogenization of renal tissues and cells was performed in the kit’s extracting solution. Following centrifugation at 8000 × *g* and 4 °C, the supernatant solution was measured at 412 nm.

### Malondialdehyde (MDA) Assay

The levels of malondialdehyde (MDA) in HK-2 cells were detected by colorimetric determination using the MDA assay kit (S0131S, Beyotime, Shanghai, China) as described. The absorbance of each microwell was read at 532 nm.

### Iron quantification and ferroOrange staining

Intracellular iron concentration was determined using an iron assay kit (ab83366, Abcam) according to the manufacturer’s instructions. For live-cell fluorescent imaging of intracellular Fe^2+^, cells were incubated for 30 min in serum-free medium supplemented with 1 µmol/L FerroOrange fluorescent probe (G1727, Servicebio). Subsequently, intracellular iron ions were visualized using a Carl Zeiss LSM 980 laser scanning confocal microscope (Tokyo, Japan).

### LDH release assay

Cell lactate dehydrogenase release was quantified to represent cell mortality. The LDH release into the incubation medium after cell membrane damage was measured using an LDH diagnostic kit (G1780, Promega) according to manufacturer’s instructions.

### Determination of NAD+/NADH ratio

NAD^+^ and NADH levels were determined by using NAD^+^/NADH kit (S0175, Beyotime) using the manufacturer’s instructions. NAD^+^ and NADH levels were read on a spectrophotometer using 450 nm as the primary wavelength.

### Propidium Iodide (PI) staining

Propidium Iodide (PI) staining was used to differentiate between dead and live cells. The PI staining was obtained by using PI kit (KGA214-10 ~ KGA214-50, KeyGEN) according to manufacturer’s instructions. After incubation at 37 °C in the dark for 30 min. and the cells were gently washed once with PBS and then observed under an Olympus BX53 microscope (Olympus, Tokyo, Japan).

### siRNA-mediated gene silencing

Cells were cultured in medium without antibiotics. Short interfering RNA (siRNA) for target genes or negative control siRNA were delivered into cells by the Lipofectamine 3000 reagent (Invitrogen, Carlsbad, CA) following the manufacturer’s protocol. siRNA targeting sequence: si-GPX4-homo: 5’-GGAGUAACGAAGAGAUCAATT-3’, 3’-UUGAUCUCUUCGUUACUCCTT-5’; si-SLC7A11-homo: 5’-GGGCUGAUUUAUCUUCGAUTT-3’, 3’-AUCGAAGAUAAAUCAGCCCTT-5’; siNC 5’-UUCUCCGAACGUGUCACGUTT-3’, 3’-ACGUGACACGUUCGGAGAATT-5’.

### Co-immunoprecipitation (co-IP) assay

HK-2 cells were lysed by the addition of 300 μL of lysis buffer containing proteinase inhibitors to 10 cm plates, followed by centrifugation at 12,000 × *g* for 15 min to obtain the supernatant. The supernatant was quantified by the BCA method. Protein A magnetic beads (B23202, Selleck) were preincubated with diluted antibodies on a rotary mixer at 4 °C overnight. Equal amounts of cell lysates obtained in the previous step were incubated with antibody-coated magnetic beads overnight at 4 °C. After three washes with lysis buffer containing protease inhibitors, the beads were eluted with 1× SDS‒PAGE sample loading buffer and boiled at 100 °C for 5 min. Following the removal of the magnetic beads, the precipitated protein complexes were subjected to Western blot analysis.

### Statistics

Data are presented as means ± standard deviation (mean ± SD). Statistical analyses were conducted using GraphPad Prism (v.8.0, GraphPad Software, San Diego, CA). The normality assumption of the data distribution was assessed via the Kolmogorov-Smirnov test. Comparisons between two groups were performed using two-tailed Student’s unpaired t-test for normally distributed data and Mann–Whitney rank sum test for non-normally distributed data. One-way ANOVA followed by post hoc Tukey’s test was employed to determine differences between multiple groups with one variable. For comparisons involving multiple groups with more than one variable, two-way ANOVA followed by post hoc Tukey’s test was utilized. The Pearson correlation coefficient was applied for correlation analysis as appropriate. Statistical significance was defined as *P < 0.05, **P < 0.01, ***P < 0.001, where *P* < 0.05 serving as the threshold for significance.

## Results

### IFI16/p204 expression was upregulated in the kidneys with renal ischemia/reperfusion injury

**Fig. 1:**
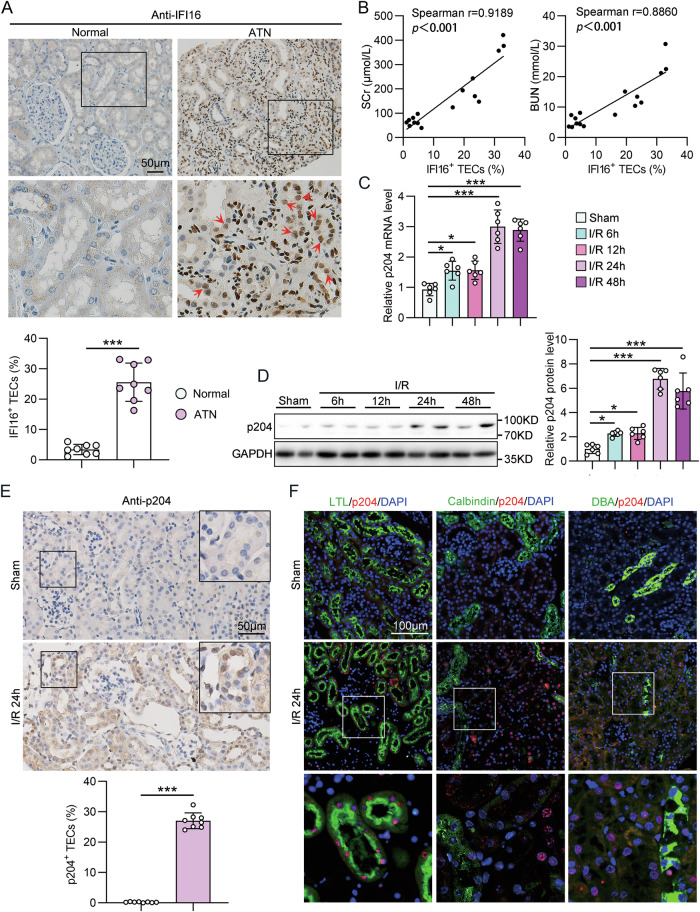
IFI16/p204 expression was upregulated in the kidneys with renal ischemia/reperfusion injury. **A** Representative photomicrographs and quantification of IFI16 immunohistochemistry in the kidneys from normal subjects (*n* = 8) and patients with ATN (*n* = 8). Red arrows indicate representative IFI16^+^ TECs. **B** Correlation between renal IFI16^+^ TECs percentage and available SCr and BUN data of normal subjects and ATN patients. **C** Relative mRNA levels of p204 in the kidneys from Sham-operated and IRI mice (*n* = 6). **D** Representative western blot gel documents and summarized data showing the protein levels of p204 in the kidneys from Sham-operated and IRI mice (*n* = 6). **E** Representative photomicrographs and quantification of p204 immunohistochemistry in the kidneys from Sham-operated and IRI mice (*n* = 8). **F** Double immunofluorescence staining for p204 and tubular segment-specific markers in the kidneys from Sham-operated and IRI mice. The following segment-specific tubular markers were used: proximal tubule, Lotus tetragonolobus lectin (LTL); distal tubule, calbindin-D28K; and collecting duct, Dolichos Biflorus Agglutinin (DBA). Data are represented as the mean ± SD. *p < 0.05, ***p < 0.001.

Immunohistochemistry (IHC) of human kidney sections indicated that the expression of IFI16 was significantly elevated in the kidneys of patients with biopsy-proven ATN compared with kidney tissues obtained from patients who underwent tumor nephrectomies without other renal diseases (Fig. 1A). Importantly, the percentage of IFI16 positive TECs was positively correlated with the serum creatinine (SCr) and blood urea nitrogen (BUN) levels of all subjects (Fig. 1B). Considering renal ischemia/reperfusion (I/R) injury (IRI) is the main cause of AKI, IRI mouse model was used to investigate the expression profile and role of p204, the murine structural and functional homolog of the human IFI16, in AKI. As shown in Fig. 1C, D, both the mRNA and protein levels of p204 in the kidney of mice were enhanced at 6 and 12 h post-I/R compared with those in Sham-operated mice and further increased at 24 and 48 h post-I/R. IHC further demonstrated that p204 was elevated predominantly in the nuclei of renal TECs of I/R-AKI mice (Fig. 1E). To further define the tubular segment specificity of p204 expression in the kidney, we used double immunofluorescence staining for p204 (red) with various tubular markers (green), including Lotus tetragonolobus lectin (LTL) for proximal tubules, Calbindin-D28K for distal tubules, and Dolichos Biflorus Agglutinin (DBA) for collecting tubules (Fig. 1F). The upregulated expression of p204 was mainly found in the proximal tubules of IRI mice (Fig. 1F).

### Tubule-specific deficiency of p204 ameliorated renal injury in IRI mice

**Fig. 2:**
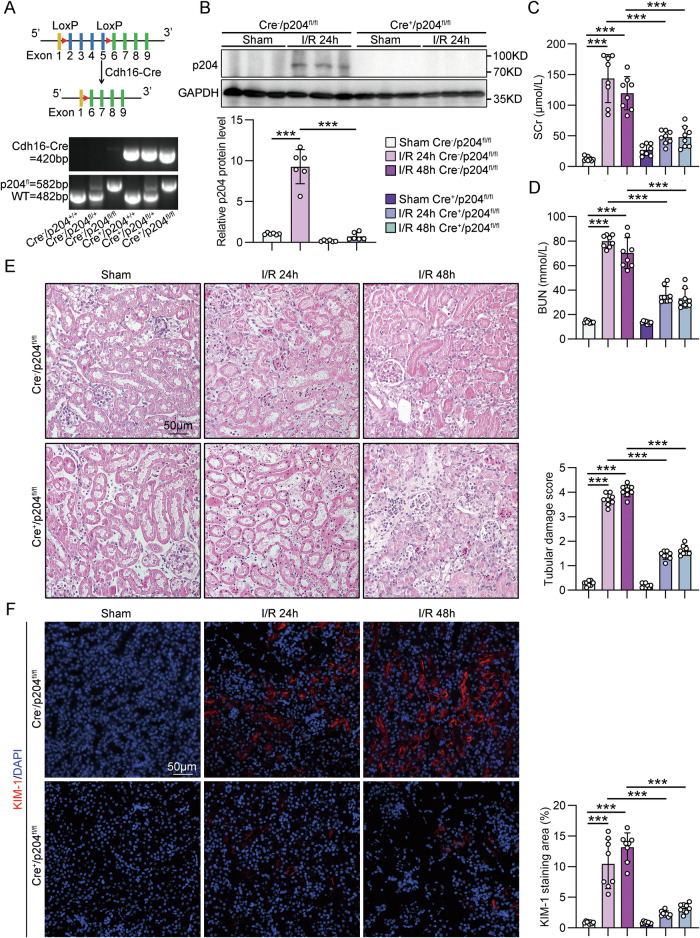
Tubule-specific deficiency of p204 ameliorated renal injury in IRI mice. **A** Experimental scheme for generating tubule-specific p204 knockout mice (Cdh16-Cre^+^/p204^fl/fl^) by using the Cre-loxP recombination system. Genotyping was confirmed by tail genotyping. Flox genotyping yielded distinct fragment sizes of 582 bp and 482 bp for the mutant and wild-type (WT) alleles, respectively. The WT genotype was characterized by a single 482 bp band, whereas the homozygous p204^fl/fl^ genotype was distinguished by a single 582 bp band. In contrast, the heterozygous p204^fl/+^ genotype exhibited both bands. Cre-positive (Cre^+^) yields a 420 bp band, but Cre-negative (Cre^−^) has no band. **B** Representative Western blot gel documents and summarized data showing the protein levels of p204 in the cortex of kidneys from different groups of IRI model mice (*n* = 6). **C** SCr levels of different groups of IRI model mice (*n* = 8). **D** BUN levels of different groups of IRI model mice (*n* = 8). E Representative photomicrographs of H&E staining and quantitative assessment of tubular damage in kidneys from different groups of IRI model mice (*n* = 8). **F** Representative photomicrographs and quantification of KIM-1 immunofluorescence staining in the kidneys from different groups of IRI model mice (*n* = 8). Data are represented as the mean ± SD. ***p < 0.001.

To investigate the role of p204 of TECs in AKI of mice, we generated tubule-specific p204-deficient mice (*Cre*^*+*^*/p204*^*fl/fl*^) by a Cre-loxP recombination system involving Cdh16-Cre, which was validated by tail genotyping and Western blot analysis (Fig. 2A, B). Although Cre^+^/p204^fl/fl^ mice exhibited a normal phenotype, with no apparent defects in kidney morphology or function, tubule-specific p204 deficiency significantly attenuated kidney injury in IRI mice. The levels of SCr and BUN of Cre^+^/p204^fl/fl^ mice were significantly reduced at 24 and 48 h post-I/R compared with those of Cre^−^/p204^fl/fl^ IRI mice (Fig. 2C, D). Hematoxylin and eosin (H&E) staining demonstrated that tubule-specific p204 deficiency ameliorated renal injury in IRI mice (Fig. 2E), which is consistent with the reduction of kidney tubule injury marker KIM-1 and NGAL (Figs. 2F and S1A). In addition, tubule-specific loss of p204 significantly inhibited renal inflammation in IRI mice, as evidenced by reduced F4/80^+^ macrophage and LY6B^+^ neutrophil infiltration and inflammatory mediator expression (Fig. S1B–D).

### IFI16 deficiency alleviated the cell death of TECs under I/R conditions

**Fig. 3:**
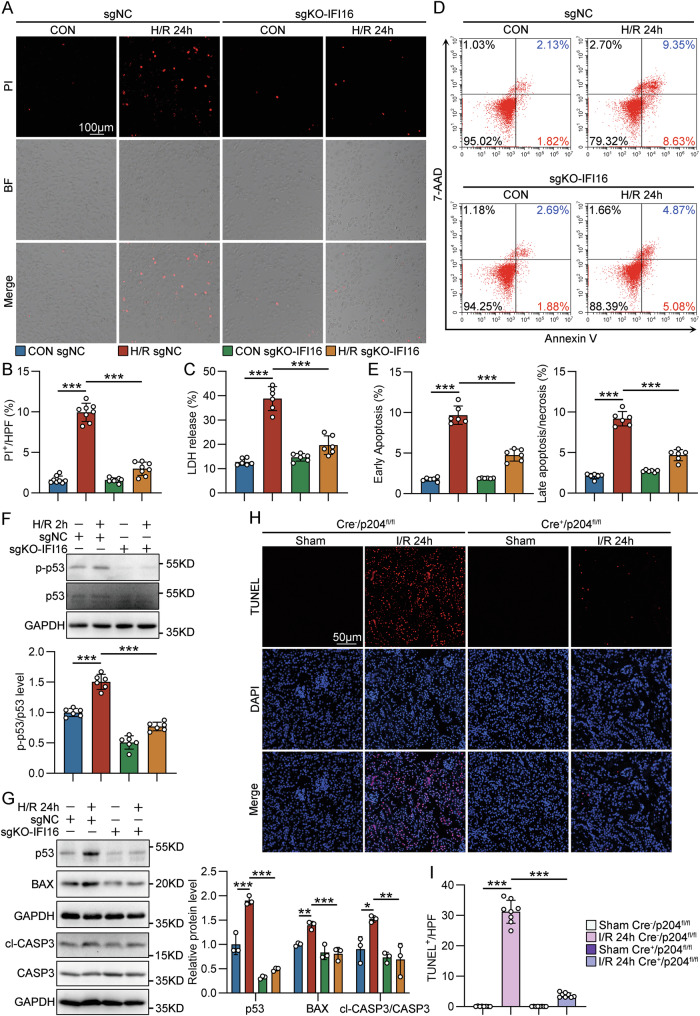
IFI16 deficiency reduced TEC cell death under I/R conditions. Representative photomicrographs (**A**) and quantification (**B**) of propidium iodide (PI) staining in sgNC- or sgKO-IFI16-transfected HK-2 cells with H/R treatment (*n* = 8). **C** The extracellular LDH levels in sgNC- or sgKO-IFI16-transfected HK-2 cells with H/R treatment (*n* = 6). Representative scatter plots of flow cytometry analysis (**D**) and quantitative data (**E**) depicting the early apoptosis (Annexin V^+^/7-AAD^−^, bottom right quadrant, percentages were highlighted in blue) and late apoptosis/necrosis (Annexin V^+^/7-AAD^+^, top right quadrant, percentages were highlighted in red) of sgNC- or sgKO-IFI16-transfected HK-2 cells with H/R treatment (*n* = 6). **F** Representative Western blot gel documents and summarized data showing the protein levels of p-p53 in sgNC- or sKO-IFI16-transfected HK-2 cells with H/R treatment (*n* = 6). **G** Representative Western blot gel documents and summarized data showing the protein levels of p53 and BAX and caspase-3 cleavage in sgNC- or sgKO-IFI16-transfected HK-2 cells with H/R treatment (*n* = 3). Representative photomicrographs (**H**) and quantification (**I**) of terminal deoxynucleotidyl transferase-mediated uridine triphosphate nick-end labeling (TUNEL) assays of the kidneys from different groups of IRI model mice to assess renal cell death (*n* = 8). Nuclei were revealed by using DAPI staining. Data are represented as the mean ± SD. *p < 0.05, **p < 0.01, ***p < 0.001.

In vitro, both the mRNA and protein levels of IFI16 were induced by hypoxia/reoxygenation (H/R) treatment in HK-2 cells (Fig. S2A, B). To determine the role of IFI16 in I/R-induced TEC injury in vitro, CRISPR/Cas9-sgRNA system was used to establish HK-2 sublines lacking IFI16 (IFI16 knockout, sgKO-IFI16) (Fig. S2C). Compared with control HK-2 sublines (sgNC), cell death induced by H/R, measured by plasma membrane disruption (positive propidium iodide [PI] staining and leakage of cytosolic enzyme lactate dehydrogenase [LDH] to the culture medium), was significantly attenuated by IFI16 KO (Fig. 3A–C). The flow cytometry using Annexin V and 7-AAD staining indicated that IFI16 KO reduced the percentage of cells undergoing early apoptosis (Annexin V^+^/7-AAD^−^) (4.670% ± 0.746% vs 9.680 ± 1.134%; *p* < 0.0001) and late apoptosis/necrosis (Annexin V^+^/7-AAD^+^) (4.686% ± 0.695% vs 9.185 ± 0.897%; *p* < 0.0001) compared with those of H/R-treated HK-2 cells (Figs. 3D, E, and Figure S2D). Meanwhile, IFI16 KO in HK-2 cells restored H/R-induced p53 phosphorylation at 2 h (Fig. 3F) and Caspse-3 cleavage, elevated p53 and Bax expression, and RIPK3 and MLKL phosphorylation at 24 h after reoxygenation (Figs. 3G and S2E). By using Terminal deoxynucleotidyl transferase (TdT) dUTP Nick-End Labeling (TUNEL) assay, a technique to detect apoptotic cells undergoing extensive DNA degradation during the late stages of apoptosis, we found that TEC-specific p204 deficiency ameliorated the apoptosis of TECs in the kidney from IRI mice (Fig. 3H, I). These data indicated that IFI16/p204 contributes to necrotic and apoptotic cell death of TECs under I/R conditions.

### IFI16/p204 mediated ferroptosis-associated lipid peroxidation in TECs during AKI

**Fig. 4:**
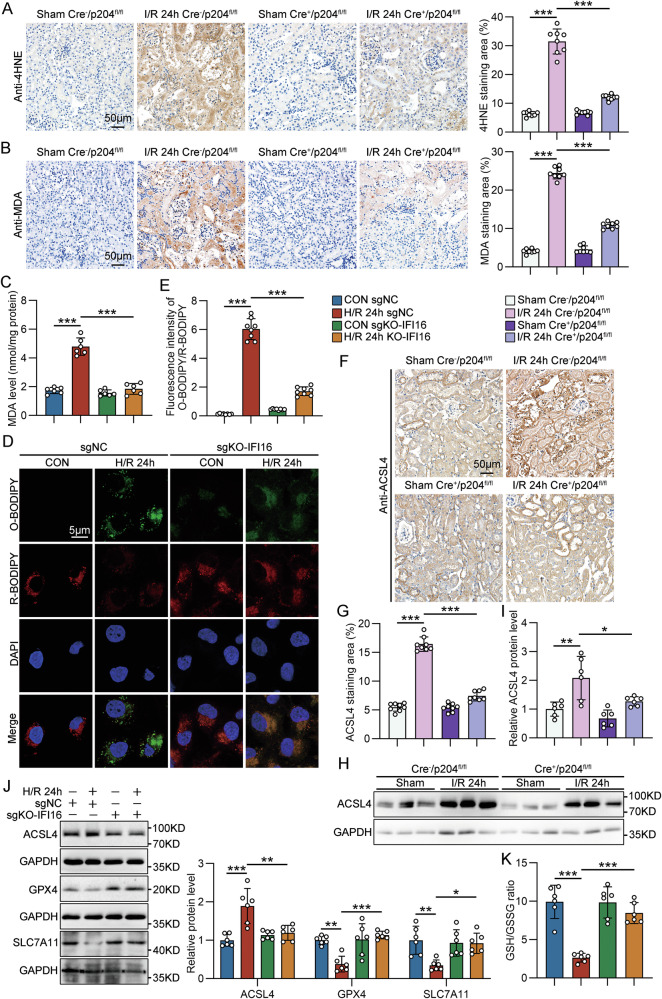
IFI16/p204 mediated ferroptosis-associated lipid peroxidation in TECs during AKI. **A** Representative photomicrographs and quantification of 4HNE immunohistochemistry in the kidneys from different groups of IRI model mice (*n* = 8). **B** Representative photomicrographs and quantification of MDA immunohistochemistry in the kidneys from different groups of IRI model mice (*n* = 8). **C** Quantitative analysis of MDA levels in sgNC- or sgKO-IFI16-transfected HK-2 cells with H/R treatment (*n* = 6). Representative photomicrographs (**D**) and quantification (**E**) of BODIPY-C11 probe staining in sgNC- or sgKO-IFI16-transfected HK-2 cells with H/R treatment (*n* = 8). Representative photomicrographs (**F**) and quantification (**G**) of ACSL4 immunohistochemistry in the kidneys from different groups of IRI model mice (*n* = 8). Representative Western blot gel documents (**H**) and summarized data (**I**) showing the protein levels of ACSL4 in the cortex of kidneys from different groups of IRI model mice (*n* = 6). **J** Representative Western blot gel documents and summarized data showing the protein levels of ACSL4, GPX4, and SLC7A11 in sgNC- or sgKO-IFI16-transfected HK-2 cells with H/R treatment (*n* = 6). **K** Quantitative analysis of GSH/GSSG levels in sgNC- or sgKO-IFI16-transfected HK-2 cells with H/R treatment (*n* = 6). Data are represented as the mean ± SD. *p < 0.05, **p < 0.01, ***p < 0.001.

In addition to promoting apoptosis, p53 also sensitizes cells to ferroptosis by downregulating SLC7A11, the core subunit of cystine/glutamate antiporter system (System Xc^−^), suggesting IFI16/p204 may play a role in ferroptosis of TECs during AKI. To verify this hypothesis, we detected the effects of IFI16/p204 on lipid peroxidation, a marker of ferroptosis, in IRI. As shown in Fig. 4A, B, the elevated levels of lipid peroxidation products 4-hydroxynonenal (4HNE) and malondialdehyde (MDA) in TECs of kidney from IRI mice were alleviated by tubule-specific p204 deficiency. MDA concentration analysis and BODIPY-C11 probe staining indicated that IFI16 KO reduced H/R-induced production of lipid peroxides in HK-2 cells (Fig. 4C–E). Acylcoenzyme A (CoA) synthetase long-chain family member 4 (ACSL4) is a key enzyme that catalyses the activation of long-chain fatty acids, facilitates polyunsaturated fatty acids (PUFAs) incorporation into membrane lipids and acts as a specific biomarker and driver of ferroptosis. The IHC and Western blot analysis revealed that tubule-specific p204 deficiency inhibited the enhancement of ACSL4 protein levels in the kidney from IRI mice (Fig. 4F–I). Consistently, IFI16 KO mitigated H/R-induced ACSL4 expression in HK-2 cells (Fig. 4J). On the other hand, our data also showed that IFI16/p204 in TECs regulates cystine/glutamate antiporter (System Xc^−^), glutathione (GSH) and glutathione peroxidase 4 (GPX4) (System Xc^−^/GSH/GPX4) axis, an important antioxidant system for ferroptosis. The decreased expression of GPX4 and SLC7A11 (the light chain of System Xc^−^) in TECs under I/R conditions was abrogated by IFI16 and p204 deficiency in vitro (Fig. 4J) and in vivo (Fig. S3A, S3B), respectively. In addition, IFI16 KO effectively restored the reduction of GSH/GSSG ratios in H/R-treated HK-2 cells (Fig. 4K). Then, the expression of GPX4 and SLC7A11 was inhibited by a short interfering RNA (siRNA) approach in sgKO-IFI16 HK-2 cells, respectively. As shown in Fig. S3C, D, either GPX4 or SLC7A11 knockdown impeded the inhibitory effect of IFI16 KO on H/R-induced ferroptosis indicated by restored MDA levels, suggesting that IFI16 exerts its pro-ferroptosis role in AKI by regulating GPX4/SLC7A11 axis. Importantly, by using inhibitors Z-VAD-FMK for apoptosis, Nec-1S for necroptosis, and Ferrostatin-1 for ferroptosis, the PI staining assay indicated that inhibition of ferroptosis greatly rescued the cell death exacerbated by IFI16-overexpression in H/R-treated HK-2 cells, while the effects of inhibiting apoptosis or necroptosis were mild, suggesting that ferroptosis is the dominant type of cell death regulated by IFI16 in TECs in response to renal I/R (Fig. S4A, B). In addition, Z-VAD-FMK, Nec-1S, or Ferrostatin-1 specifically inhibited H/R-induced apoptosis, necroptosis, and ferroptosis in IFI16-overexpressing HK-2 cells, respectively (Fig. S4C, D). These results indicate that inhibition of IFI16-enhanced ferroptosis does not lead to compensatory activation of apoptotic and necrotic cell death in vitro.

### IFI16/p204 participated in the imbalance of iron homeostasis in TECs during AKI

**Fig. 5:**
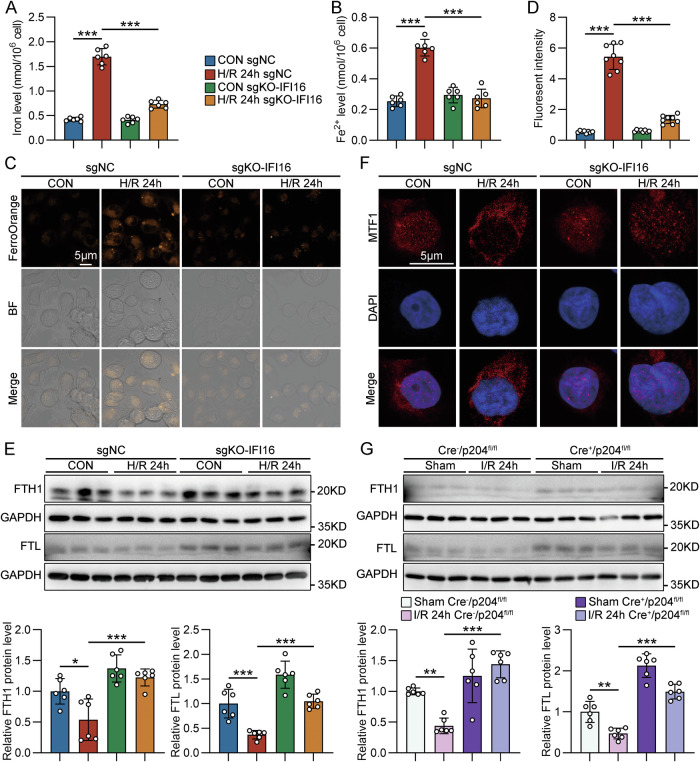
IFI16/p204 participated in the imbalance of iron homeostasis in TECs during AKI. Quantitative analysis of iron (**A**) and Fe^2+^ (**B**) levels in sgNC- or sgKO-IFI16-transfected HK-2 cells with H/R treatment (*n* = 6). Representative photomicrographs (**C**) and quantification (**D**) of FerroOrange staining in sgNC- or sgKO-IFI16-transfected HK-2 cells with H/R treatment (*n* = 8). FerroOrange (Orange) exhibited the localization of intracellular free iron in living cells. **E** Representative Western blot gel documents and summarized data showing the protein levels of FTH1 and FTL in sgNC- or sgKO-IFI16-transfected HK-2 cells with H/R treatment (*n* = 6). **F** The subcellular localization of MTF1 in sgNC- or sgKO-IFI16-transfected HK-2 cells with H/R treatment (*n* = 8). Representative photomicrographs are shown to visualize the subcellular locations of MTF1 upon different groups. **G** Representative western blot gel documents and summarized data showing the protein levels of FTH1 and FTL in the cortex of kidneys from different groups of IRI model mice (*n* = 6). Data are represented as the mean ± SD. *p < 0.05, **p < 0.01, ***p < 0.001.

Ferroptosis is intimately associated with iron metabolism due to the role of iron in mediating the production of reactive oxygen species and enzyme activity in lipid peroxidation. Therefore, we further detected the effects of IFI16/p204 on iron metabolism in TECs under I/R conditions. As shown in Fig. 5A–D, H/R treatment triggered an increase of intracellular iron and ferrous ions in HK-2 cells, which was effectively alleviated by IFI16 KO. Balance of iron homeostasis depends on the expression levels and activities of iron carriers, iron transporters, and iron regulatory and storage proteins. Here, we found that IFI16 KO in HK-2 cells restored the expression of iron-regulated factors related to iron storage (ferritin heavy and light chains, FTH1 and FTL) (Fig. 5E). Metal-regulated transcription factor 1 (MTF1), a transcription factor in eukaryotes that binds to metal response elements (MREs) to promote gene transcription that maintains metal homeostasis. MTF1 was found in both nuclei and cytosol of HK-2 cells and H/R treatment mainly restricted MTF1 protein in the cytosol. However, IFI16 KO enhanced the nuclear localization of MTF1 in H/R-treated HK-2 cells (Fig. 5F). In vivo, TEC-specific p204 deficiency enhanced the protein levels of FTH1 and FTL in the kidney from mice with IRI (Figs. 5G and S4E).

### IFI16/p204 contributed to ferroptosis of TECs under I/R conditions through amplifying DNA damage response

**Fig. 6:**
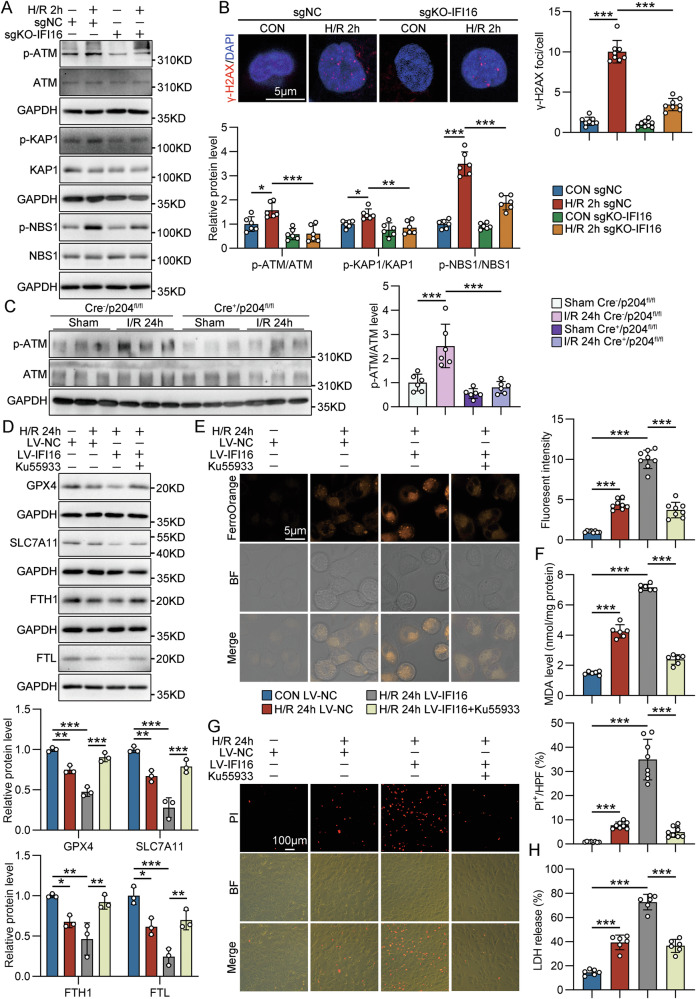
IFI16/p204 amplified DNA damage response in TECs under I/R conditions and contributed to ferroptosis. **A** Representative Western blot gel documents and summarized data showing the protein levels of p-ATM, p-KAP1 and p-NBS1 in sgNC- or sgKO-IFI16-transfected HK-2 cells with H/R 2 h treatment (*n* = 6). **B** Representative photomicrographs and quantification of γ-H2AX immunofluorescence staining in sgNC- or sgKO-IFI16-transfected HK-2 cells with H/R treatment (*n* = 8). **C** Representative Western blot gel documents and summarized data showing the protein levels of p-ATM in the cortex of kidneys from different groups of IRI model mice (*n* = 6). **D** Representative Western blot gel documents and summarized data showing the protein levels of GPX4, SLC7A11, FTH1 and FTL in LV-IFI16-GFP-transfected HK-2 cells with H/R 24 h treatment with or without Ku-55933 (10 µM) pretreatment (*n* = 3). **E** Representative photomicrographs and quantification of FerroOrange staining in LV-IFI16-GFP-transfected HK-2 cells with H/R 24 h treatment with or without Ku-55933 pretreatment (*n* = 8). **F** Quantitative analysis of MDA levels in LV-IFI16-GFP-transfected HK-2 cells with H/R 24 h treatment with or without Ku-55933 pretreatment (*n* = 6). **G** Representative photomicrographs and quantification of propidium iodide (PI) staining in LV-IFI16-GFP-transfected HK-2 cells with H/R 24 h treatment with or without Ku-55933 pretreatment (*n* = 8). **H** The extracellular LDH levels in LV-IFI16-GFP-transfected HK-2 cells with H/R 24 h treatment and with or without Ku-55933 pretreatment (*n* = 6). Data are represented as the mean ± SD. *p < 0.05, **p < 0.01, ***p < 0.001.

IFI16 has been identified as an amplifier for DDR, which not only can signal repair processes but is also intimately involved in cell fate decisions [27]. The Western blot assay indicated that H/R stimulated the phosphorylation of ataxia-telangiectasia mutated (ATM) and its downstream substrates, such as NBS1 and KAP1 in HK-2 cells at 2 h after reoxygenation, and was attenuated by IFI16 KO (Fig. 6A). At the same time, the number of phosphorylated H2AX (γ-H2AX) foci in H/R-treated HK-2 cells was effectively inhibited by IFI16 KO (Fig. 6B). In vivo, tubule-specific p204 deficiency inhibited the levels of phosphorylated ATM in the kidneys from IRI mice (Fig. 6C). A kinome screen study has identified ATM as a determinant of ferroptosis through regulating labile iron levels [17]. In line with this finding, our data indicated that ATM inhibition restored the reduced expression of GPX4 (0.903 ± 0.055 vs 0.477 ± 0.057; *p* < 0.0001), SLC7A11 (0.789 ± 0.080 vs 0.278 ± 0.123; *p* = 0.0003), FTH1 (0.920 ± 0.085 vs 0.464 ± 0.201; *p* = 0.0058), and FTL (0698 ± 0.121 vs 0.245 ± 0.103; *p* = 0.0041) (Fig. 6D) in H/R-treated HK-2 cells with IFI16 overexpression, and in turn prevented ferrous iron accumulation (Fig. 6E), lipid peroxidation (Fig. 6F), and cell death (Fig. 6G, H).

### IFI16 bound with and activated PARP-1 in TECs with H/R treatment

**Fig. 7:**
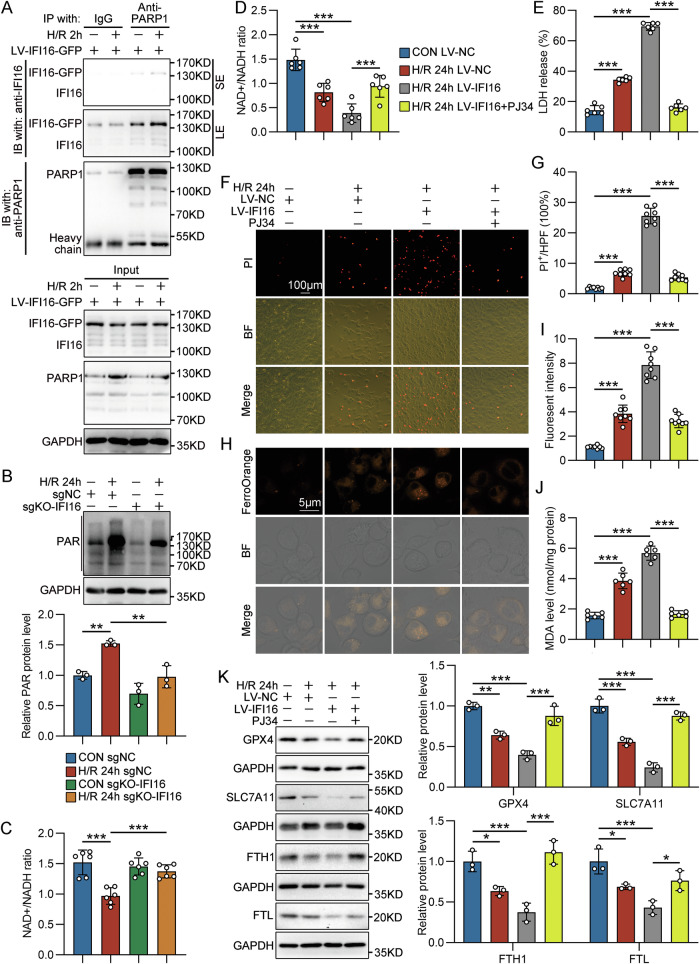
IFI16 bound with and activated PARP-1 in TECs with H/R treatment. **A** Representative Western blot gel documents of the co-immunoprecipitation (co-IP) assay detecting the interaction between IFI16 and PARP1 in HK-2 cells overexpressing IFI16-GFP with H/R 2 h treatment. SE short exposure, LE long exposure. **B** Representative Western blot gel documents and summarized data showing the levels of protein PARylation in sgNC- or sgKO-IFI16-transfected HK-2 cells with H/R 24 h treatment (*n* = 3). **C** Quantitative analysis of NAD^+^/NADH levels in sgNC- or sgKO-IFI16-transfected HK-2 cells with H/R 24 h treatment (*n* = 6). **D** Quantitative analysis of NAD^+^/NADH levels in LV-IFI16-GFP-transfected HK-2 cells with H/R 24 h treatment with or without PJ34 (10 µM) pretreatment (*n* = 6). **E** The extracellular LDH levels in LV-IFI16-GFP-transfected HK-2 cells with H/R 24 h treatment with or without PJ34 pretreatment (*n* = 6). Representative photomicrographs (**F**) and quantification (**G**) of propidium iodide (PI) staining in LV-IFI16-GFP-transfected HK-2 cells with H/R 24 h treatment with or without PJ34 pretreatment (*n* = 8). Representative photomicrographs (**H**) and quantification (**I**) of FerroOrange staining in LV-IFI16-GFP-transfected HK-2 cells with H/R 24 h treatment with or without PJ34 pretreatment (*n* = 8). **J** Quantitative analysis of MDA levels in LV-IFI16-GFP-transfected HK-2 cells with H/R 24 h treatment with or without PJ34 pretreatment (*n* = 6). **K** Representative Western blot gel documents and summarized data showing the protein levels of GPX4, SLC7A11, FTH1 and FTL in LV-IFI16-GFP-transfected HK-2 cells with H/R 24 h treatment with or without PJ34 pretreatment (*n* = 3). Data are represented as the mean ± SD. *p < 0.05, **p < 0.01, ***p < 0.001.

Poly(ADP-ribose) polymerase 1 (PARP-1) is widely recognized as a first-line responder molecule in DDR. Here, the results of co-immunoprecipitation (co-IP) assay demonstrated that PARP-1 binds with IFI16-GFP and endogenous IFI16 in HK-2 cells overexpressing IFI16-GFP, which was enhanced in response to H/R treatment for 2 h (Fig. 7A). Knockout of IFI16 in HK-2 cells inhibited H/R-induced protein PolyADP ribosylation (PARylation) (Fig. 7B) and oxidized nicotinamide adenine dinucleotide (NAD^+^) depletion (Fig. 7C). Importantly, the pretreatment with PARP inhibitor PJ34 effectively reduced IFI16 overexpression-aggravated NAD^+^ consumption, LDH leakage, PI positive rate, ferrous iron accumulation, and lipid peroxidation in HK-2 cells treated with H/R (Fig. 7D–K), suggesting that IFI16-mediated ferroptosis of TECs under I/R conditions is dependent on the activation of PARP-1.

### IFI16-amplified DDR and ferroptosis were dependent on its HIN and PYRIN domains in H/R-treated HK-2 cells

**Fig. 8:**
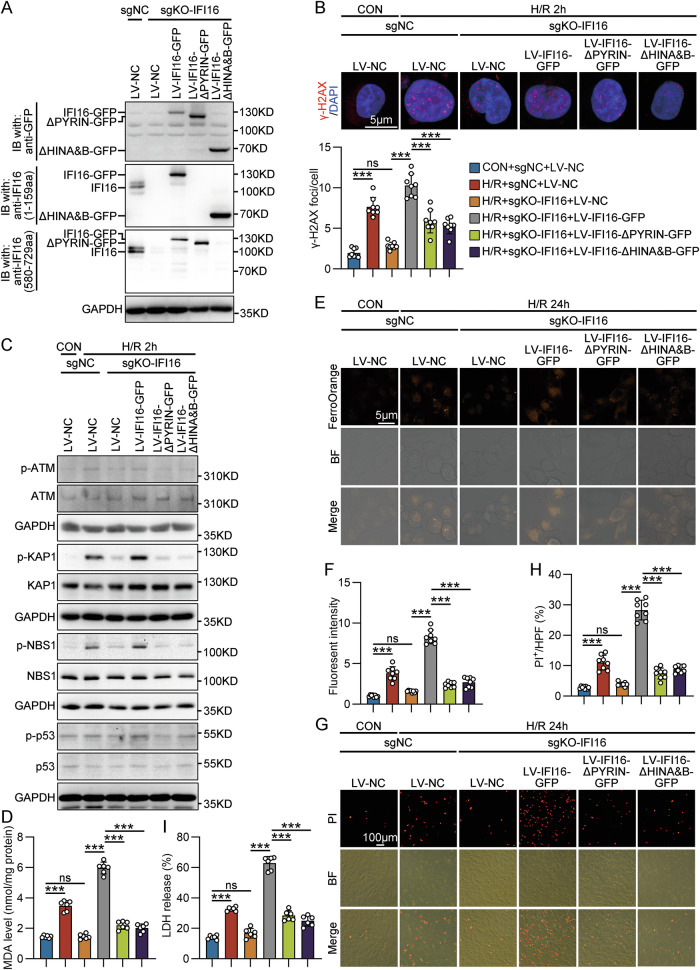
IFI16-amplified DDR and ferroptosis were dependent on its HIN and PYRIN domains in H/R-treated HK-2 cells. **A** Western blot analysis showing the expression of GFP-tagged IFI16 and IFI16 bearing PYRIN or HINA&B domain deletions in sgKO-IFI16 HK-2 cells transduced with lentivirus coding wild-type IFI16-GFP, IFI16-ΔPYRIN-GFP mutant, or IFI16-ΔHINA&B-GFP mutant. An anti-GFP antibody was used to detect GFP-tagged proteins. IFI16 antibody (1G7) targeting the N-terminus (1-159aa) was used to detect endogenous IFI16, wild-type IFI16-GFP, and IFI16-ΔHINA&B-GFP mutant. IFI16 antibody targeting the C-terminus (580-729aa) was used to detect endogenous IFI16, wild-type IFI16-GFP, and IFI16-ΔPYRIN-GFP mutant. **B** Representative photomicrographs and quantification of γ-H2AX immunofluorescence staining in different IFI16-mutant HK-2 cells with H/R 2 h treatment (*n* = 8). **C** Representative Western blot gel documents showing the protein levels of p-ATM, p-KAP1, p-NBS1, and p-p53 in different IFI16-mutant HK-2 cells with H/R 2 h treatment. **D** Quantitative analysis of MDA levels in different IFI16-mutant HK-2 cells with H/R 24 h treatment (*n* = 6). Representative photomicrographs (**E**) and quantification (**F**) of FerroOrange staining in different IFI16-mutant HK-2 cells with H/R 24 h treatment (*n* = 8). Representative photomicrographs (**G**) and quantification (**H**) of propidium iodide (PI) staining in different IFI16-mutant HK-2 cells with H/R 24 h treatment (*n* = 8). **I** The extracellular LDH levels in different IFI16-mutant HK-2 cells with H/R 24 h treatment (*n* = 6). Data are represented as the mean ± SD. ***p < 0.001, ns not significant.

IFI16 is a predominantly nuclear protein, our data showed that IFI16 is mainly located in the nucleolus of HK-2 cells stable expressing GFP-tagged IFI16 (IFI16-GFP) under normal conditions, and partially enters the nucleoplasm and forms foci after H/R treatment (Fig. S5A). By using HK-2 cells expressing IFI16-GFP or linker histone H1.2 (H1.2-GFP), a laser microirradiation resulted in the eviction of H1.2 from microirradiated sites, where IFI16 is rapidly accumulated (Fig. S5B), suggesting that IFI16 may bind to damaged DNA. IFI16 composes a PYRIN domain involved mainly in protein-protein interactions and two HIN domains mediating DNA binding. To map IFI16 domain(s) necessary for its interaction with PARP1, we constructed GST-tagged IFI16 and IFI16 mutants bearing different domain deletions (IFI16-ΔPYRIN, IFI16-ΔHINA, IFI16-ΔHINB, and IFI16-ΔHINA&B), and tested them by GST pull-down assay. The GST, GST-tagged IFI16 and its mutants immobilized on anti-GST magnetic beads were incubated with recombinant human His-PARP1, respectively. Western blot analysis revealed that only IFI16-ΔPYRIN failed to bind with His-PARP1, demonstrating that PYRIN domain of IFI16 is necessary for IFI16-PARP1 interaction (Fig. S6A–D). We next investigated the domain(s) in IFI16 responsible for activating DDR in response to H/R treatment. IFI16-KO HK-2 cells were transduced with lentivirus coding wild-type IFI16-GFP, IFI16-ΔPYRIN-GFP mutant, or IFI16-ΔHINA&B-GFP mutant, and their successful expression was confirmed by western blot and GFP fluorescence imaging (Figs. 8A and S6E). Our results showed that IFI16-KO-reduced DNA damage and DDR activation in H/R-treated HK-2 cells were restored by re-expression of wild-type IFI16, but not by IFI16-ΔPYRIN and IFI16-ΔHINA&B mutants, indicating that both HIN and PYRIN domains are required for IFI16 to amplify DDR (Figs. 8B, C and S6F). As expected, re-expression of IFI16-ΔPYRIN and IFI16-ΔHINA&B mutants in sgKO-IFI16 HK-2 cells failed to mediate ferroptosis, apoptosis and necrosis in response to H/R treatment (Figs. 8D–I and S6G, H).

## Discussion

Available preclinical studies have suggested an important role of ferroptosis in AKI. However, the activation mechanism of this novel regulated cell death in AKI is still unclear. Our study provides compelling multi-level evidence to establish a critical pathogenic axis linking DNA damage to ferroptosis through IFI16-amplyfied DDR. We demonstrate that targeting IFI16 can attenuate ferroptosis of TECs and ameliorate kidney dysfunction, providing new insights into the pathophysiology and intervention of AKI.

One of the most important findings of this study is that we identify IFI16/p204 as a master regulatory hub of ferroptosis that determines cell fate of TECs in response to DNA damage in I/R-AKI. As the immediate-early sensors in the DDR, ATM and ATR are unquestionably pro-survival by promoting DNA repair and cell cycle arrest [28]. However, in instances of high double-strand breaks (DSB) levels, ATM and ATR switch off repair and stimulate cell death [28, 29]. Although a study for triple-negative breast cancer suggested that IFI16 inhibits DNA repair by binding to histone-evicted DNA at damaged DNA sites [30], IFI16 has long been regarded as an amplifier of DDR potentiating the ATM-p53 signaling and maximizing the p53-mediated transcription [26, 27, 31, 32], which is consistent with our findings that IFI16/p204 facilitates the hyperactivation of ATM-p53 DDR pathway in TECs under I/R conditions. Lipid peroxidation and intracellular iron accumulation are two central biochemical events leading to ferroptosis. A line of evidence has emerged that ATM is indispensable for ferroptosis by dominating the intracellular labile iron pool. Inhibition or genetic ablation of ATM enhances the expression of iron storage- and export-associated genes, and reduces ferritinophagy receptor NCOA4 phosphorylation, in turn restrain ferritinophagy for ferritin degradation [17, 33]. As a downstream target of ATM, p53 is tightly associated with all ferroptosis-associated key metabolic pathways. In most cases, p53 promotes ferroptosis by coordinating multiple downstream pathways, such as p53/SLC7A11/GPX4 and p53/YAP1/ACSL4 [34]. In line with this, we demonstrate that IFI16 limits GSH production by activating the ATM-p53 pathway and increases intracellular labile iron in an ATM-dependent manner, leading to TEC ferroptosis in I/R-AKI. A recent study identified IFI16 as a key gene associated with radioresistance, which attenuates irradiation-induced ferroptosis in glioblastoma multiforme [35]. The heterogeneity of IFI16 in regulating ferroptosis may be related to the differences in cell types across different diseases. Compared to normal cells, tumor cells exhibit aberrant redox homeostasis and higher levels of reactive oxygen species (ROS). To counteract oxidative burden, tumor cells increase their antioxidant status to optimize ROS-driven proliferation, while at the same time avoiding ROS thresholds that would trigger senescence, apoptosis, or ferroptosis [36]. Another difference is the status of p53, the most altered gene in human cancers. Interestingly, U251 and Ln229 cell lines used to demonstrate the anti-ferroptotic role of IFI16 contain p53 mutations [37, 38], whereas normal HK-2 cells have a basic expression of wild-type p53 [39]. The differences in redox and p53 status between tumor cells and normal cells may determine the opposite roles of IFI16 in regulating ferroptosis in tumors and AKI. IFI16 may alter BRCA1-mediated DNA damage repair and the extent of DNA damage by binding to BRCA1. Further investigation is needed to determine whether the interaction between IFI16 and BRCA1 in TECs is weaker than that in tumor cells, leading to excessive DDR activation rather than DNA repair. Of note, a recent report reveals that BRCA1 has a dual role in regulating ferroptosis. BRCA1 deficiency in breast cancer cells promotes cellular resistance to erastin-induced ferroptosis but sensitizes cancer cells to ferroptosis induced by GPX4 inhibitors [40], suggesting the possible pathways through which IFI16 promotes or inhibits ferroptosis. In addition, cancer-associated fibroblasts and tumor-associated macrophages in the tumor microenvironment also contribute to tumor cell ferroptosis resistance through paracrine signaling in certain cancer types [41].

The binding and activation of PARP-1 by IFI16 is the basis of the IFI16-DDR-ferroptosis axis. To our knowledge, this is the first discovery of the binding between IFI16 and PARP-1. PARP-1 is an essential regulator of ATM activation. Upon DNA damage, linker histone H1.2 undergoes rapid PARP1-dependent chromatin dissociation through PARylation and further proteasomal degradation, resulting in ATM activation [42]. Our observations that inhibition of PARP-1 disrupts ATM activation, lipid peroxidation and intracellular iron accumulation in H/R-treated IFI16-overexpressing HK-2 cells indicate that IFI16 conduces to PARP-1-dependent DDR and subsequent ferroptosis in TECs in I/R-AKI. As a first-line responder molecule in DDR, PARP-1-catalyzed poly-ADP ribosylation of itself and target proteins relies on the consumption of NAD^+^ and leads to depletion of ATP pools, ultimately triggering regulated necrosis, referred to as parthanatos [43, 44]. NAD^+^ depletion is considered as a key driver of metabolic dysfunction in AKI, linking energy deficits to poor clinical outcomes [45]. An increasing body of studies have demonstrated that PARP-1-dependent parthanatos contributes to the pathogenesis of I/R-AKI by using genetic knockout models or chemical inhibitors of PARP-1 [46, 47].

The multi-domain architecture and unique recognition mechanisms of IFI16 enable it to serve as a multifunctional player with the ability to sense DNA damage, detect viral threats, and regulate inflammatory responses [48]. The PYRIN domain of IFI16 has been reported to mediate protein-protein interaction, such as STING [23], ASC [49], and BRCA1 [26] in certain cell types, and is also involved in IFI16 oligomerization upon binding to DNA [50]. Consistently, our data reveals that the PYRIN domain of IFI16 mediates the interaction between IFI16 and PARP1. IFI16 HIN domains are responsible for recognizing and binding to viral DNA, damaged host DNA, or even self-DNA released [51, 52]. In addition, IFI16 HIN-A domain binding to p53 results in enhanced DNA binding of p53 and increased transcriptional activation [53]. Our data shows that IFI16-mediated DDR-ferroptosis axis is dependent on both the PYRIN and HIN domains, suggesting that both PARP1-binding and damaged DNA-binding abilities of IFI16 are involved in cell fate decisions of TECs in I/R-AKI. However, our study has limitations in mapping the IFI16-binding domain(s) on PARP1. Further efforts are warranted to elucidate the specific mechanism underlying the interaction between IFI16 and PARP-1.

Another important issue is that IFI16/p204-regulated cell death opens new avenues for therapeutic strategies of I/R-AKI. In our work, we clearly demonstrate that IFI16-mediated PARP-1-ATM-p53 pathway hyperactivation contributes to multiple cell death modalities, including ferroptosis, apoptosis and necroptosis. Among them, ferroptosis is the dominant type of cell death regulated by IFI16 in TECs in response to renal I/R. Evidence from interventional preclinical studies suggests that when different cell death pathways are activated simultaneously, inhibition of one pathway will not prevent cell death but rather may change the modality of cell death due to the multiple layers of interconnections existing between different modalities of regulated cell death, including shared triggers, molecular components and protective mechanisms [10]. For example, pan-caspase inhibition shifted the mode of cell death of tubular cells from apoptosis to necroptosis in AKI [54, 55]. Similar interconnections have also been described between apoptosis and pyroptosis [56], and between necroptosis and ferroptosis in AKI [57], illustrating the potential dangers of interfering with individual cell death pathway. Pan-cell death inhibitors targeting two or three cell death pathways may increase the likelihood of success [57–59]. However, combination therapy may exacerbate the safety concerns [10]. On the other hand, emerging evidence has shown that pharmacological and genetic inhibition of DDR components, such as ATM and ATR, do not ameliorate, but instead exacerbate I/R-AKI and cisplatin-induced AKI [12, 60, 61], suggesting the requirement of moderate DDR-mediated DNA repair for the survival of injured TECs and the adverse effects of excessive suppression of DDR on AKI. In this scenario, IFI16 is likely to be a better and more reasonable therapeutic target for AKI that can eliminate the hyperactivated DDR and simultaneously inhibit apoptosis, necrosis, and ferroptosis by reducing IFI16 expression or disrupting its DNA binding, PARP1 binding or oligomerization activities.

Collectively, these findings demonstrate for the first time that IFI16/p204 facilitates the hyperactivation of PARP-1-ATM-p53 DDR pathway, leading to ferroptosis of TECs in I/R-AKI. Our findings extend the knowledge linking DNA damage and ferroptosis in pathophysiology and advance the mechanisms determining cell fate of TECs in AKI. Modulation of IFI16-mediated hyperactivation of DDR may provide a novel approach for the treatment of AKI.

## Supplementary information

Supplemental Figures and Supplemental Tables

The uncropped original images of electrophoretic blots and gels
