# Supplementary material for: IFI16 is essential to linking DNA damage and ferroptosis in acute kidney injury
Source: Cell Death Dis. 2026 Mar 23;17(1):350. doi: 10.1038/s41419-026-08604-5 (PMC13039746; doi:10.1038/s41419-026-08604-5)
Supplement: Supplementary file 1 — Supplemental Figures and Supplemental Tables [file 41419_2026_8604_MOESM1_ESM.docx]

**Supplemental Information**


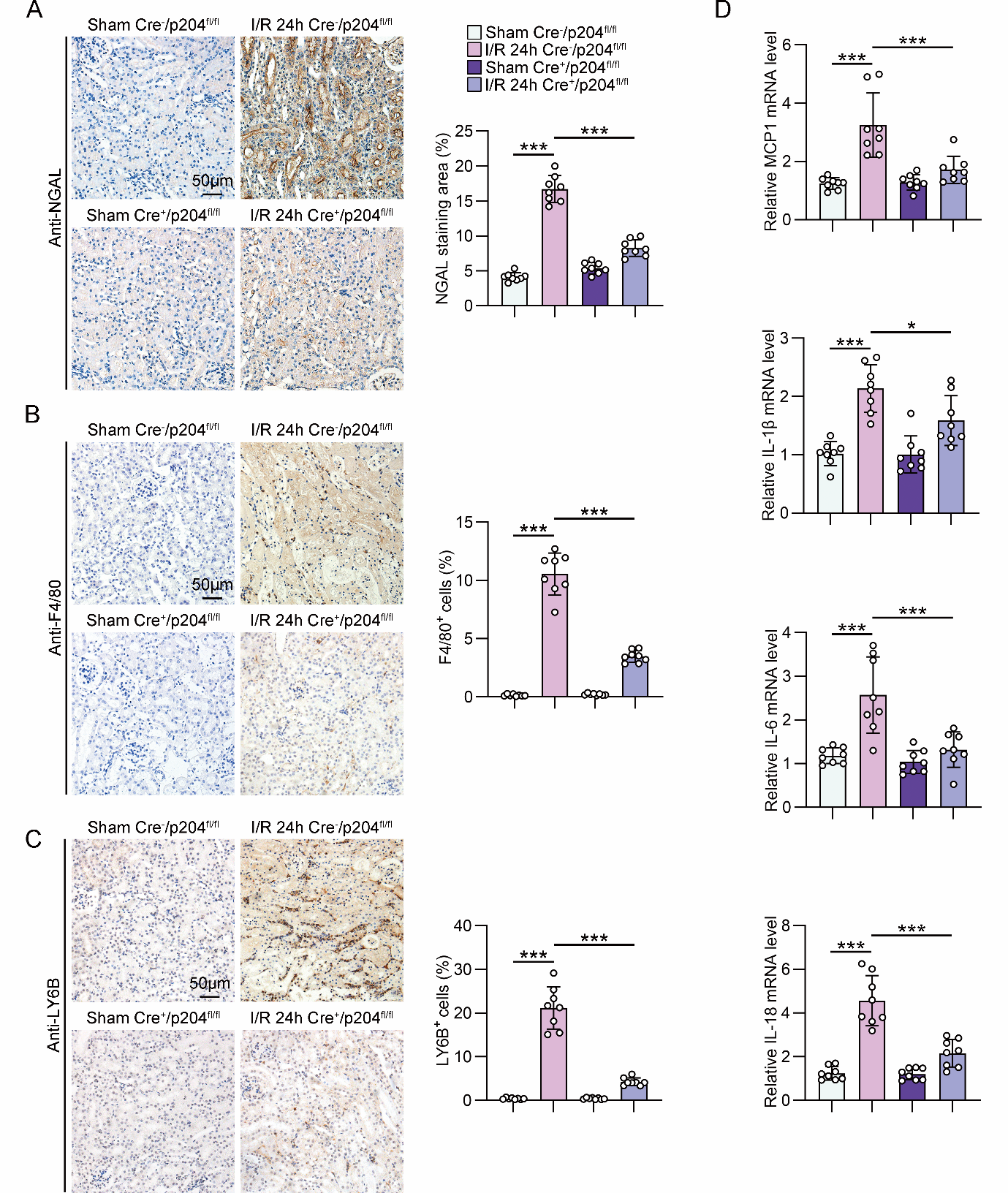
**Supplemental Figures**

**Figure S1. Tubule-specific deficiency of p204 in mice ameliorated renal inflammation in IRI mice.**

(A) Representative photomicrographs and quantification of NGAL immunohistochemistry in the kidneys from different groups of IRI model mice (n = 8). (B) Representative photomicrographs and quantification of F4/80 immunohistochemistry in the kidneys from different groups of IRI model mice (n = 8). (C) Representative photomicrographs and quantification of LY6B immunohistochemistry in the kidneys from different groups of IRI model mice (n = 8). (D) Relative mRNA levels of MCP1, IL-1β, IL-6, IL-18 in the kidneys from different groups of IRI model mice (n = 8). Data are represented as the mean ± SD. *p < 0.05, ***p < 0.001.


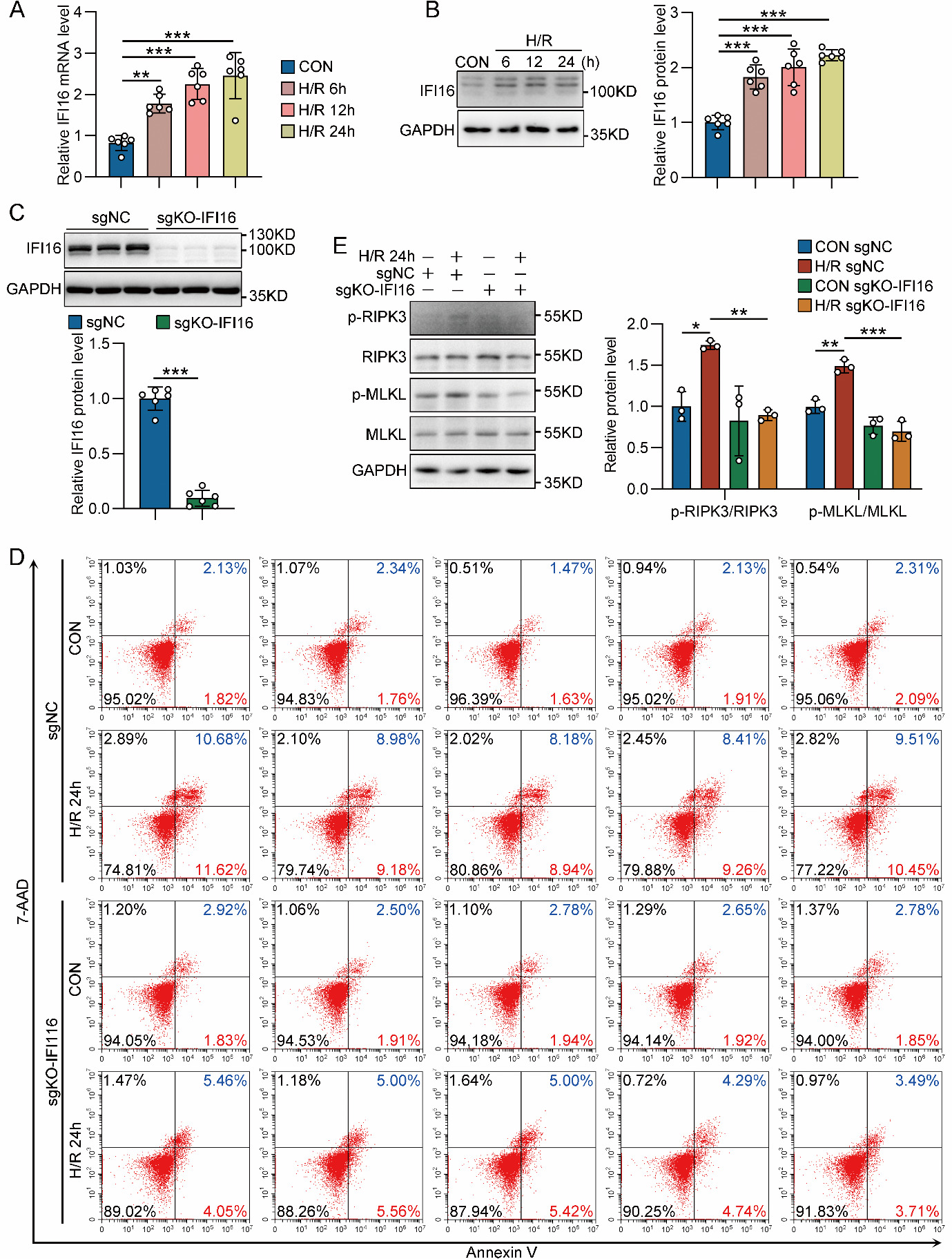


**Figure S2. IFI16 knockout inhibited Hypoxia/reoxygenation (H/R)-induced apoptotic and necrotic cell death of HK-2 cells.**

(A) Relative mRNA levels of IFI16 in HK-2 cells with H/R treatment (n = 6). (B) Representative western blot gel documents and summarized data showing the protein levels of IFI16 in HK-2 cells with H/R treatment (n = 6). (C) Representative western blot gel documents and summarized data showing the protein levels of IFI16 in sgNC or sgKO-IFI16 HK-2 cells (n = 6). (D) The flow cytometry scatter plots of other five independent replications depicting the early apoptosis (Annexin V^+^/7-AAD^-^, bottom right quadrant, percentages were highlighted in blue) and late apoptosis/necrosis (Annexin V^+^/7-AAD^+^, top right quadrant, percentages were highlighted in red) of sgNC or sgKO-IFI16 HK-2 cells with H/R treatment. (E) Representative Western blot gel documents and summarized data showing the protein levels of p-RIPK3 and p-MLKL in sgNC or sgKO-IFI16 HK-2 cells with H/R treatment (n = 3). Data are represented as the mean ± SD. *p < 0.05, **p < 0.01, ***p < 0.001.


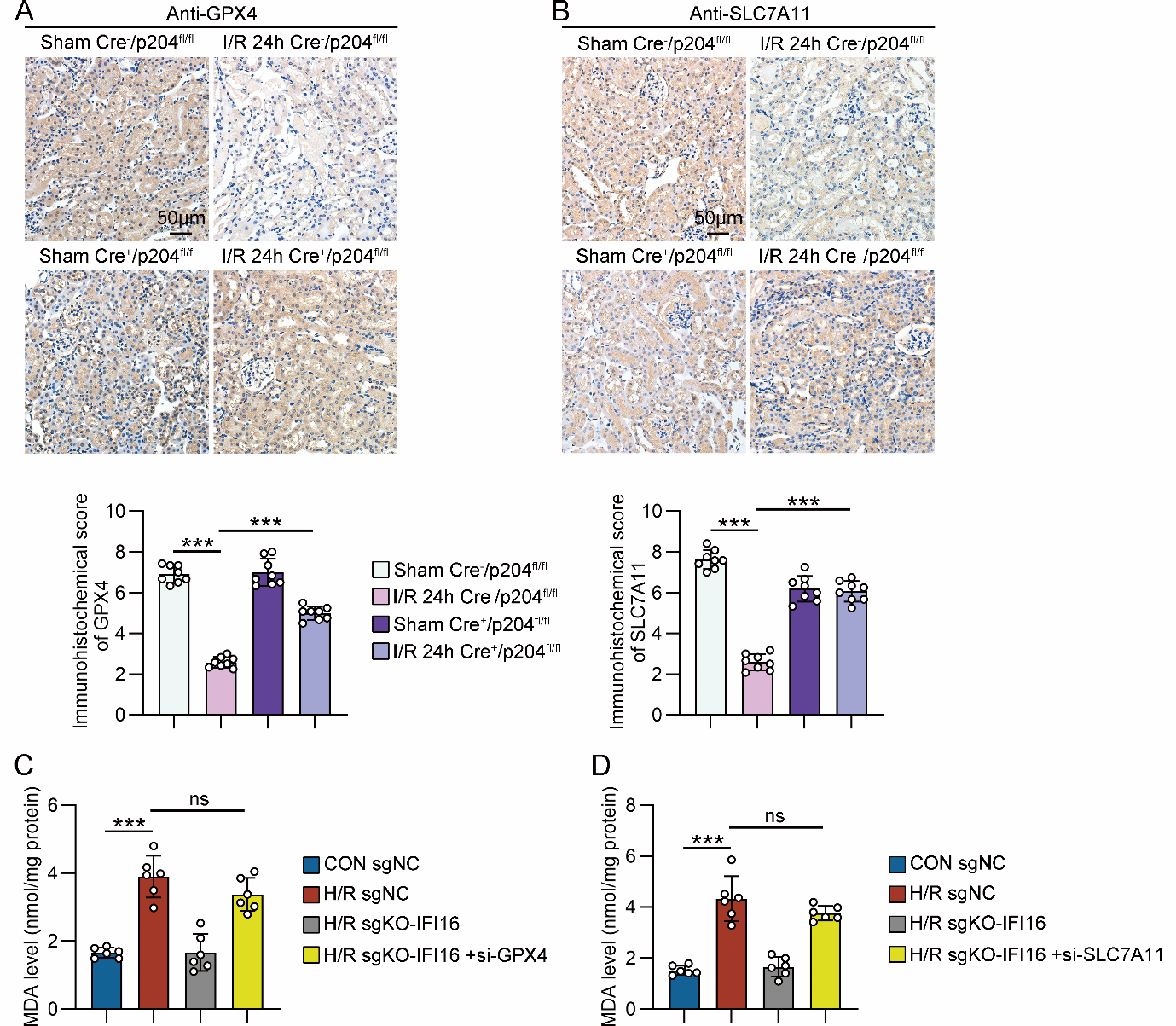


**Figure S3. Tubule-specific deficiency of p204 in mice ameliorated ferroptosis-associated lipid peroxidation in IRI mice.**

(A) Representative photomicrographs and quantification of GPX4 immunohistochemistry in the kidneys from different groups of IRI model mice (n = 8). (B) Representative photomicrographs and quantification of SLC7A11 immunohistochemistry in the kidneys from different groups of IRI model mice (n = 8). (C) Quantitative analysis of MDA levels in sgKO-IFI16 HK-2 cells with or without si-GPX4 transfection at 24h after H/R treatment (n = 6). (D) Quantitative analysis of MDA levels in sgKO-IFI16 HK-2 cells with or without si-SLC7A11 transfection at 24h after H/R treatment (n = 6). Data are represented as the mean ± SD. ***p < 0.001, ns, not significant.


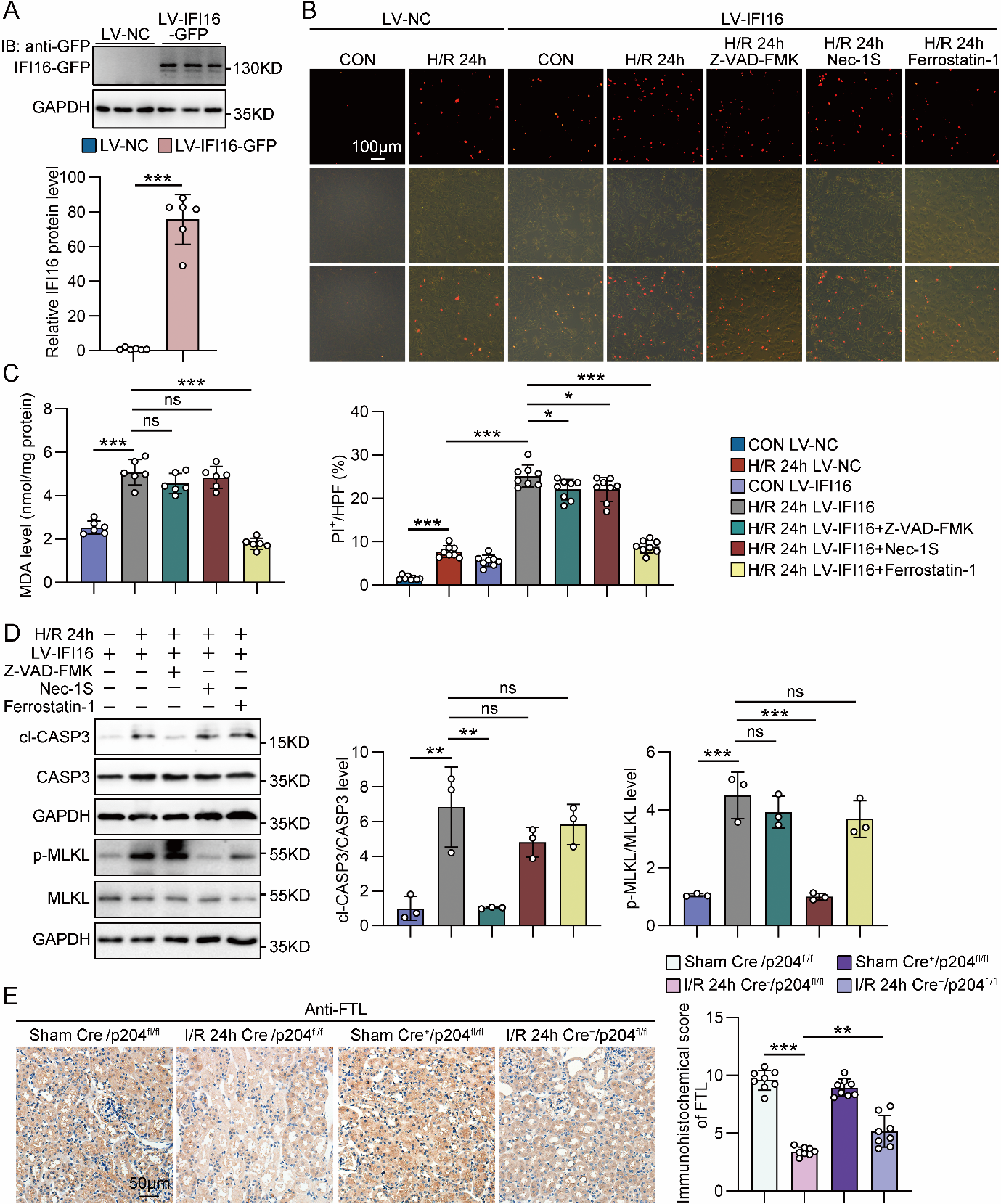


**Figure S4. Ferroptosis is the dominant type of cell death regulated by IFI16 in tubular epithelial cells in response to renal I/R.**

(A) Representative western blot gel documents and summarized data showing the protein levels of IFI16 in HK-2 cells after LV-NC or LV-IFI16-GFP lentivirus vector transduction (n = 6). (B) Representative photomicrographs and quantification of propidium iodide (PI) staining in LV-IFI16-tranduced HK-2 cells with H/R treatment in the presence of apoptosis inhibitor Z-VAD-FMK, necroptosis inhibitor Nec-1S, and ferroptosis inhibitor Ferrostatin-1 (n = 8). (C) Quantitative analysis of MDA levels in LV-IFI16-tranduced HK-2 cells with H/R treatment in the presence of Z-VAD-FMK, Nec-1S, and Ferrostatin-1 (n = 6). (D) Representative western blot gel documents and summarized data showing caspase-3 cleavage and MLKL phosphorylation in LV-IFI16-tranduced HK-2 cells with H/R treatment in the presence of Z-VAD-FMK, Nec-1S, and Ferrostatin-1 (n = 3). (E) Representative photomicrographs and quantification of FTL immunohistochemistry in the kidneys from different groups of IRI model mice (n = 8). Data are represented as the mean ± SD. *p < 0.05, **p < 0.01, ***p < 0.001, ns, not significant.


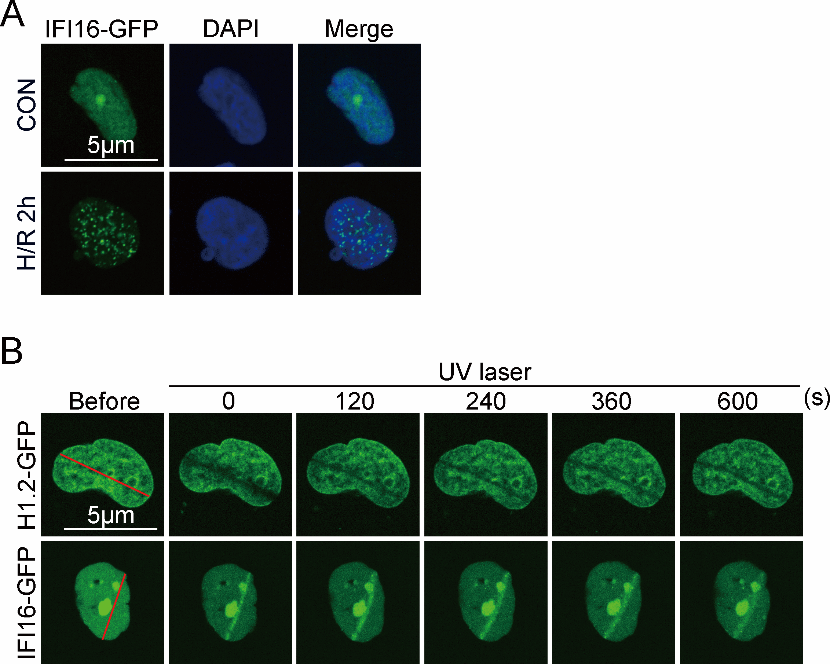


**Figure S5. The subcellular distribution of IFI16-GFP in HK-2 cells in response to H/R or laser microirradiation treatment.**

(A) Representative fluorescence images of IFI16-GFP in HK-2 cells with H/R treatment. (B) Representative fluorescence images of IFI16-GFP and H1.2-GFP in HK-2 cells with laser microirradiation.


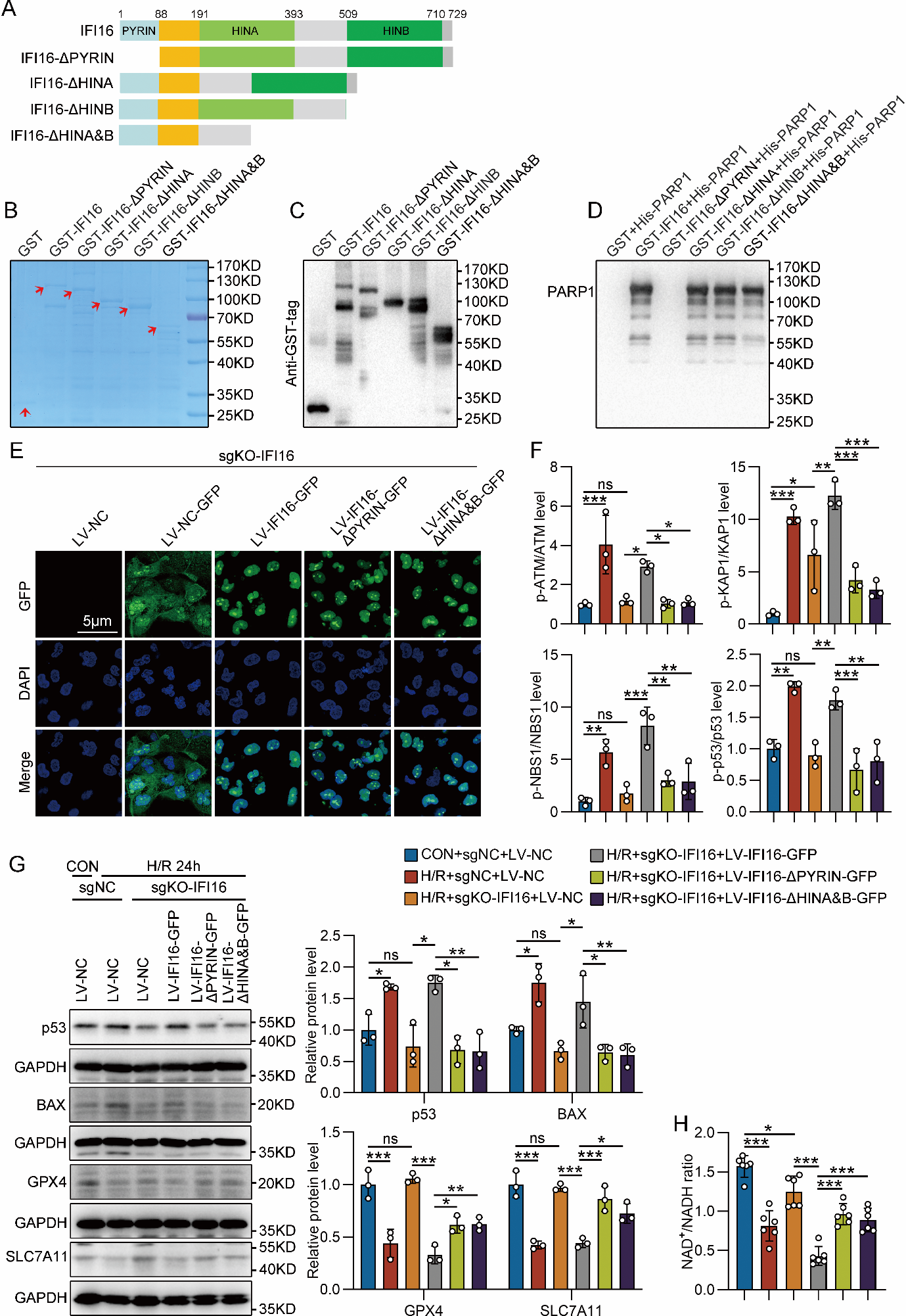


**Figure S6. IFI16-amplified cell death was dependent on its HIN and PYRIN domains in H/R-treated HK-2 cells.**

(A) Graph illustrating construction of IFI16, IFI16-ΔPYRIN IFI16-ΔHINA, IFI16-ΔHINB, and IFI16-ΔHINA&B. (B) Representative coomassie brilliant blue staining image of GST-tagged IFI16 and its mutants immobilized on anti-GST magnetic beads. Red arrows pointed to the bands of GST-tagged proteins that match the predicted molecular weight. (C) Representative western blot gel document detecting GST-tagged IFI16 and its mutants immobilized on anti-GST magnetic beads by using an antibody against GST-tag. (D) Representative western blot gel document detecting the binding between His-PARP1 and GST-tagged proteins. (E) Representative GFP fluorescence images of sgKO-IFI16 HK-2 cells transduced with negative control lentivirus (LV-NC), lentivirus coding GFP, wild-type IFI16-GFP, IFI16-ΔPYRIN-GFP mutant, or IFI16-ΔHINA&B-GFP mutant. (F) Summarized data showing the protein levels of p-ATM, p-KAP1, p-NBS1 and p-p53 in different IFI16-mutant HK-2 cells with H/R 2h treatment (n = 3). (G) Representative western blot gel documents and summarized data showing the protein levels of p53, BAX, GPX4, and SLC7A11 in different IFI16-mutant HK-2 cells with H/R 24h treatment (n = 3). (H) Quantitative analysis of NAD^+^/NADH levels in different IFI16-mutant HK-2 cells with H/R 24h treatment (n = 6). Data are represented as the mean ± SD. *p < 0.05, **p < 0.01, ***p < 0.001, ns, not significant.

**Supplemental Tables**

**Table S1. Clinical characteristics in the normal human control subjects or subjects with acute tubular necrosis.**

| **Normal human control subjects (n=8)** | | | | | | | |
| --- | --- | --- | --- | --- | --- | --- | --- |
| Number | Age  (yr) | Sex | SCr  (μmol/L) | BUN  (mmol/L) | ACR  (mg/24h) | α1-MG  (mg/L) | Complications |
| 1 | 21 | F | 62 | 3.6 | NA | NA | RN |
| 2 | 32 | M | 81 | 6.3 | NA | NA | RN |
| 3 | 27 | M | 97 | 8.1 | NA | NA | SRC |
| 4 | 38 | M | 48 | 3.4 | NA | NA | RN |
| 5 | 40 | F | 61 | 4.6 | NA | NA | RN |
| 6 | 55 | M | 39 | 3.7 | NA | NA | RN |
| 7 | 47 | M | 72 | 7.4 | NA | NA | RN |
| 8 | 24 | M | 59 | 4.4 | NA | NA | RN |
| **Subjects with tubular necrosis (n=8)** | | | | | | | |
| Number | Age  (yr) | Sex | SCr  (μmol/L) | BUN  (mmol/L) | ACR  (mg/24h) | α1-MG  (mg/L) | Complications |
| 1 | 42 | M | 357 | 21.2 | NA | NA | NS |
| 2 | 18 | M | 243 | 10.4 | NA | NA | AGN |
| 3 | 63 | M | 124 | 7.5 | NA | NA | NS |
| 4 | 40 | M | 194 | 15.1 | NA | NA | ANCA-GA |
| 5 | 56 | F | 147 | 11.5 | NA | NA | AKI |
| 6 | 40 | F | 170 | 13.4 | NA | NA | AKI |
| 7 | 43 | M | 421 | 30.74 | NA | NA | ARF |
| 8 | 52 | M | 377 | 22.5 | NA | NA | CRI |

ANCA-GA, ANCA-associated glomerulonephritis; ACR, albumin creatinine ratio; AGN, acute glomerulonephritis; AKI, acute kidney injury; ARF, acute renal failure; α1-MG, α-1 microglobulin; BUN, blood urea nitrogen; CRI, chronic renal insufficiency; F, female; M, male; NA, not available; NS, nephrotic syndrome; RN, renal neoplasms; SCr, serum creatinine; SRC, simple renal cyst.

**Table S2. Primers for tail PCR genotyping and real-time RT-PCR.**

| **GENE** | **Species** | **Primer sequences** |
| --- | --- | --- |
| *p204-Loxp* | Mouse | Forward: GGGAGATTGTGAAGCCATAGAGC  Reverse: CCTCGAGGGACCTAATAACTTCG |
| *Cdh16-Cre* | Mouse | Forward: GCCTGCATTACCGGTCGATGC  Reverse: CAGGGTGTTATAAGCAATCCC |
| *p204* | Mouse | Forward: TGGTGGGGAGTGGAAAATGG  Reverse: CCTTAGCAGGCTCCTTTGGT |
| *IFI16* | Human | Forward: ATGGATGTAGTGGGGACAGGA  Reverse: GGGGTCATTGTTTCTCGGGT |
| *MCP1* | Mouse | Forward: ACCACCTCAAGCACTTCTGT  Reverse: TAAGGCATCACAGTCCGAGT |
| *IL-1β* | Mouse | Forward: GTGTCTTTCCCGTGGACCTT  Reverse: AATGGGAACGTCACACACCA |
| *IL-6* | Mouse | Forward: CTTCTTGGGACTGATGCTGGT  Reverse: CTCTGTGAAGTCTCCTCTCCG |
| *IL-18* | Mouse | Forward: TCAAAGTGCCAGTGAACCCC  Reverse: GGTCACAGCCAGTCCTCTTAC |
| *β-actin* | Mouse | Forward: GGCTGTATTCCCCTCCATCG  Reverse: CCAGTTGGTAACAATGCCATGT |
| *β-actin* | Human | Forward: CTCACCATGGATGATGATATCGC  Reverse: AGGAATCCTTCTGACCCATGC |

**Table S3. Antibodies used in this study.**

| **Primary antibodies** | **Host** | **Dilution and supplier** | **Product ID** | **Application** |
| --- | --- | --- | --- | --- |
| p204 | Rabbit | 1:1000 for WB, 1:50 for IHC; Biorbyt, Cambridge, MA | orb539919 | WB/IHC |
| p204 | Rabbit | 1:50; ABclonal, Wuhan, China | A14002 | IF |
| GAPDH | Rabbit | 1:10000; ABclonal, Wuhan, China | A19056 | WB |
| IFI16 (1G7)  (Immunogen domain: 1-159aa) | Mouse | 1:50 for WB; 1:50 for IHC; Santa Cruz, Dallas, TX | sc-8023 | WB/IHC |
| IFI16  (Immunogen domain: 580-729aa) | Rabbit | 1:1000; ProteinTech Group, Chicago, IL | 29280-1-AP | WB |
| Lotus tetragonolobus lectin (LTL), Biotinylated |  | 1:400; Vector Labs, Burlingame, CA | B-1325-2 | IF |
| Dolichos Biflorus Agglutinin (DBA), Biotinylated |  | 1:400; Vector Labs, Burlingame, CA | B-1035-5 | IF |
| Calbindin-D28K | Mouse | 1:3000; Santa Cruz, Dallas, TX | sc-365360 | IF |
| KIM-1 | Rabbit | 1:50; Boster, Wuhan, China | BA3536 | IF |
| p-p53  (Ser15) | Rabbit | 1:1000; Cell Signaling Technology,  Boston, MA | 9284S | WB |
| p53 | Rabbit | 1:1000; Cell Signaling Technology,  Boston, MA | 2524S | WB |
| BAX | Rabbit | 1:1000; Cell Signaling Technology,  Boston, MA | 2772T | WB |
| 4HNE | Rabbit | 1:50; Abcam, Cambridge, MA | ab46545 | IHC |
| MDA | Mouse | 1:50; Abcam, Cambridge, MA | ab243066 | IHC |
| ACSL4 | Rabbit | 1:1000 for WB; 1:50 for IHC; ProteinTech Group, Chicago, IL | 22401-1-AP | WB/IHC |
| GPX4 | Mouse | 1:1000 for WB; 1:50 for IHC; ProteinTech Group, Chicago, IL | 67763-1-Ig | WB/IHC |
| SLC7A11 | Rabbit | 1:1000 for WB; 1:50 for IHC; Cell Signaling Technology, Boston, MA | 12691T | WB/IHC |
| p-ATM  (Ser1981) | Rabbit | 1:1000; Cell Signaling Technology,  Boston, MA | 5883S | WB |
| ATM | Rabbit | 1:1000; ProteinTech Group,  Chicago, IL | 27156-1-AP | WB |
| p-KAP1  (Ser824) | Rabbit | 1:1000; Cell Signaling Technology,  Boston, MA | 4127S | WB |
| KAP1 | Rabbit | 1:1000; Cell Signaling Technology,  Boston, MA | 4123S | WB |
| p-P95/NBS1  (Ser343) | Rabbit | 1:1000; Cell Signaling Technology,  Boston, MA | 3001S | WB |
| P95/NBS1 | Rabbit | 1:1000; Cell Signaling Technology,  Boston, MA | 14956S | WB |
| γ-H2AX  (Ser139) | Mouse | 1:50; Merck Millipore, Billerica, MA | 05-636 | IF |
| MTF1(H-6) | Mouse | 1:50; Santa Cruz, Dallas, TX | sc-365090 | IF |
| FTH1 | Rabbit | 1:1000; Cell Signaling Technology,  Boston, MA | 4393S | WB |
| FTL | Rabbit | 1:1000 for WB; 1:50 for IHC; ProteinTech Group, Chicago, IL | 10727-1-AP | WB/IHC |
| GFP | Rabbit | 1:1000; Abcam, Cambridge, MA | ab290 | WB |
| PARP1 | Rabbit | 1:1000; Cell Signaling Technology,  Boston, MA | 9532S | WB |
| PAR | Rabbit | 1:1000; Cell Signaling Technology,  Boston, MA | 83732S | WB |
| NGAL | Mouse | 1:50; Santa Cruz, Dallas, TX | sc-515876 | IHC |
| F4/80 | Rabbit | 1:50; Cell Signaling Technology,  Boston, MA | 70076S | IHC |
| LY6B | Rat | 1:200; Bio-Rad, Hercules, CA | MCA771G | IHC |
| Caspase-3 | Rabbit | 1:1000; Abcam, Cambridge, MA | ab32351 | WB |
| p-RIP3  (Ser227) | Rabbit | 1:1000; Cell Signaling Technology,  Boston, MA | 93654S | WB |
| RIPK3 | Rabbit | 1:1000; ProteinTech Group,  Chicago, IL | 17563-1-AP | WB |
| p-MLKL  (Ser358) | Rabbit | 1:1000; Cell Signaling Technology,  Boston, MA | 91689S | WB |
| MLKL | Mouse | 1:1000; ProteinTech Group,  Chicago, IL | 66675-1-Ig | WB |
| GST-tag | Mouse | 1:2000; ProteinTech Group,  Chicago, IL | 66001-2-Ig | WB |
