# Supplementary figures and images for: IFI16 is essential to linking DNA damage and ferroptosis in acute kidney injury
Source: Cell Death Dis. 2026 Mar 23;17(1):350. doi: 10.1038/s41419-026-08604-5 (PMC13039746; doi:10.1038/s41419-026-08604-5)

**The uncropped original images of electrophoretic blots and gels**


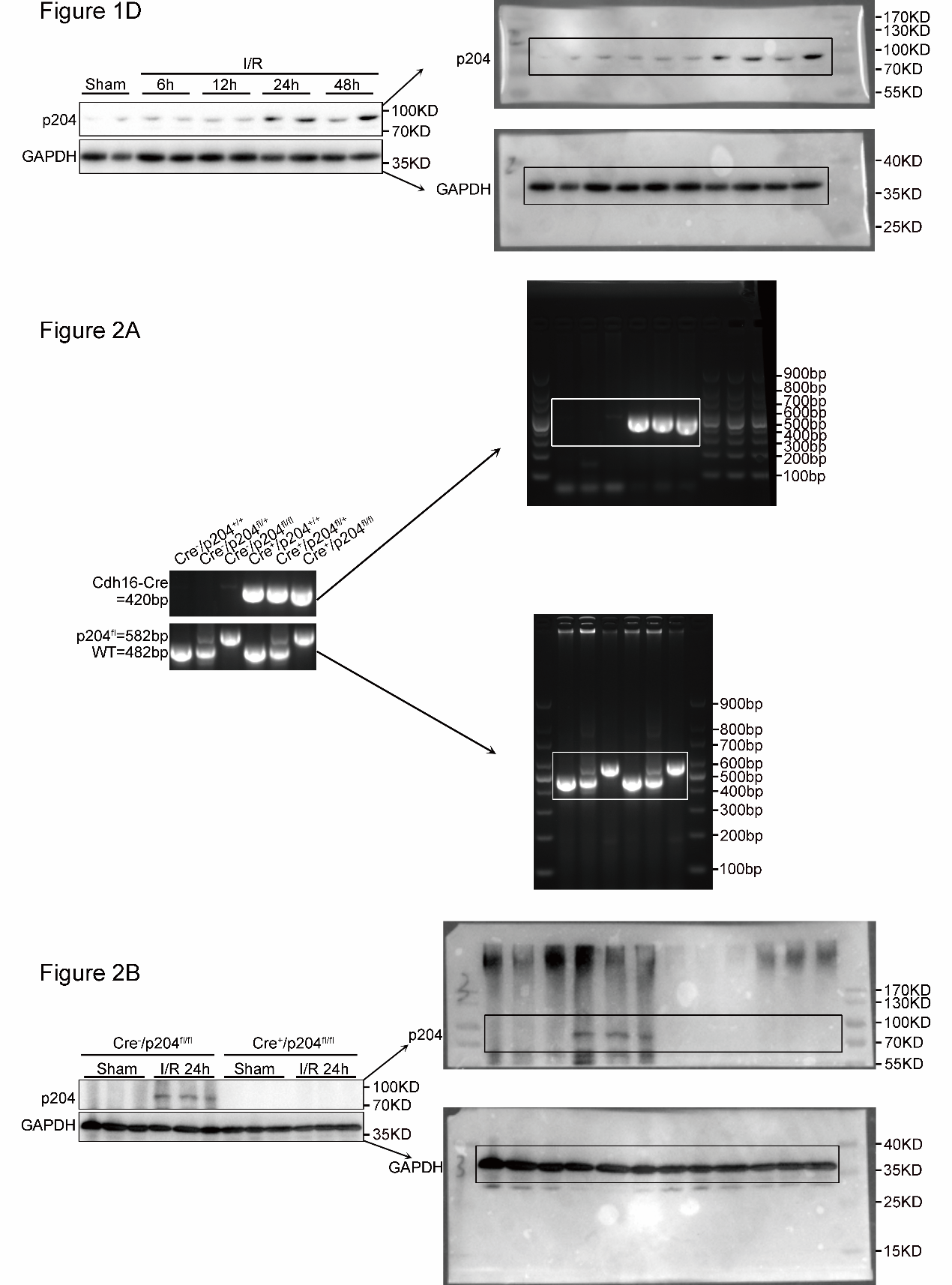


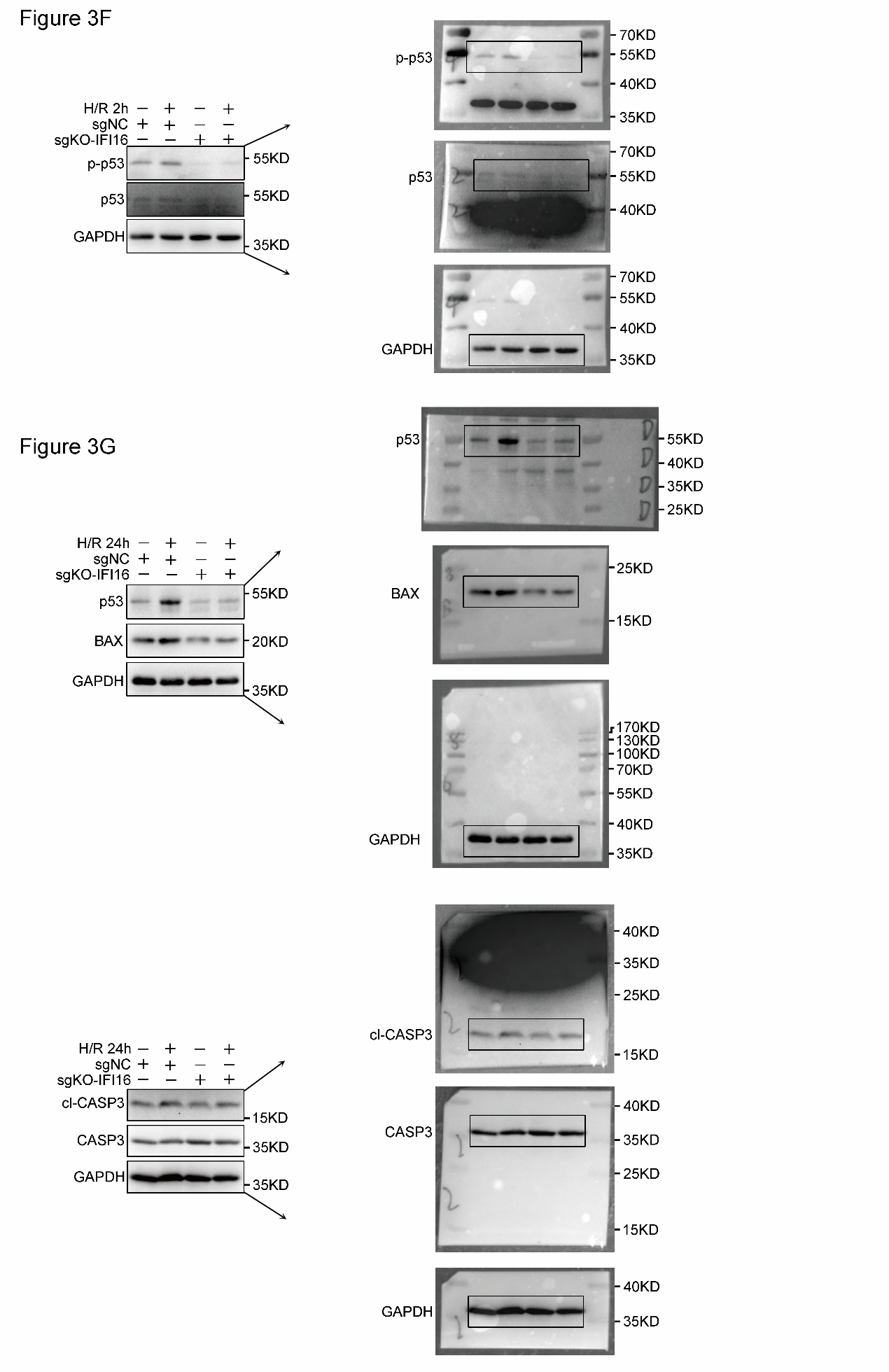


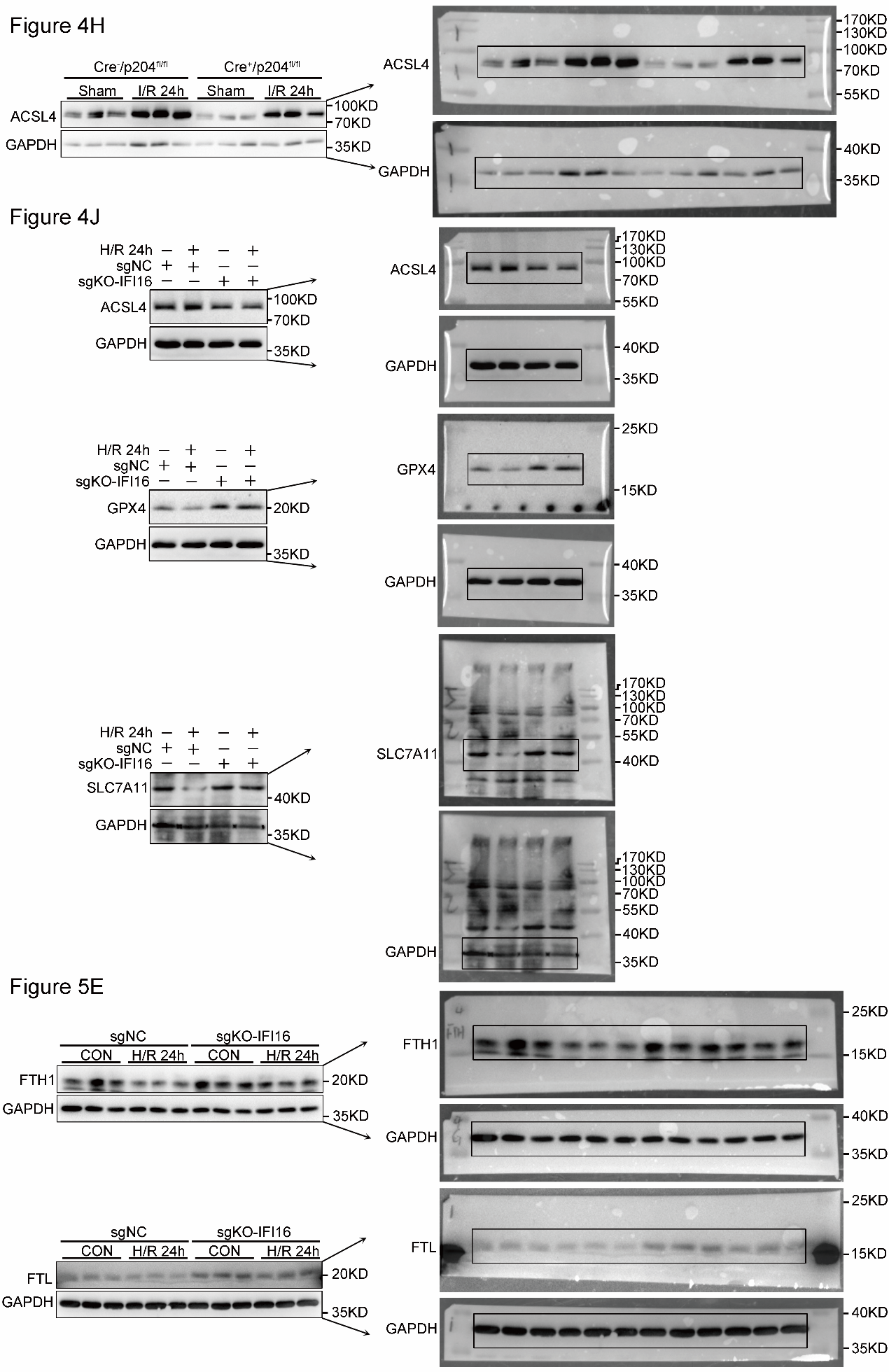


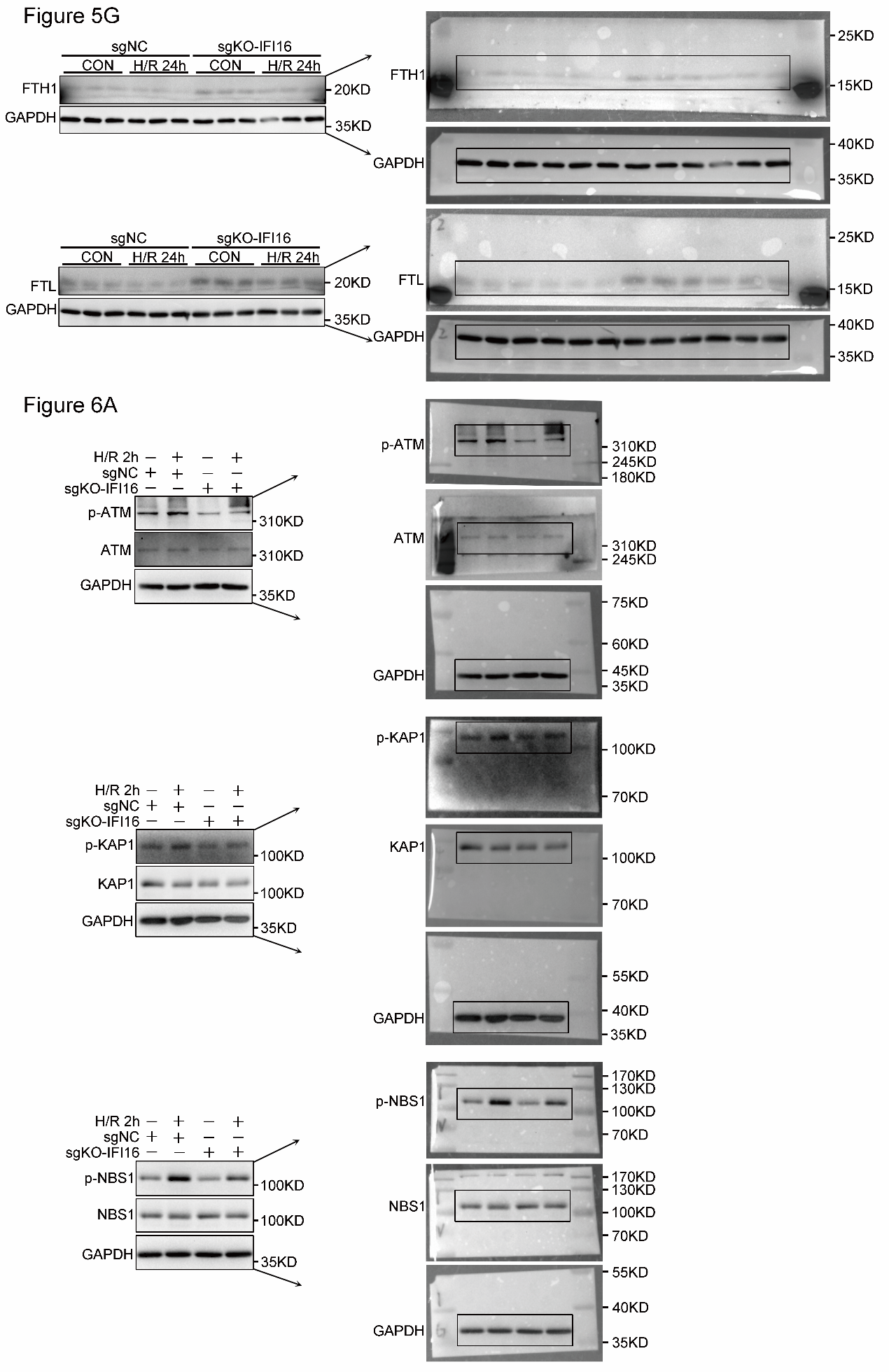


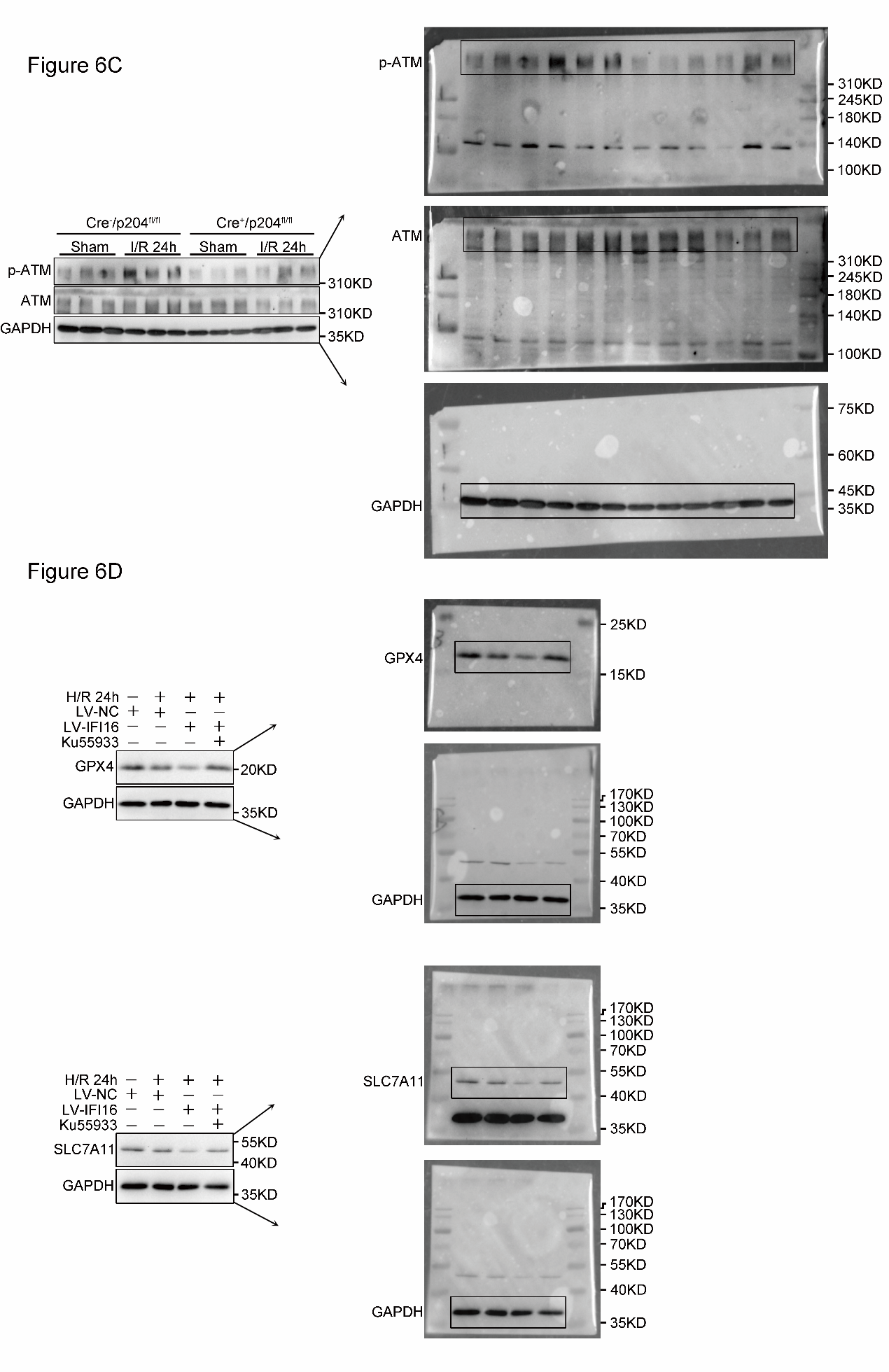


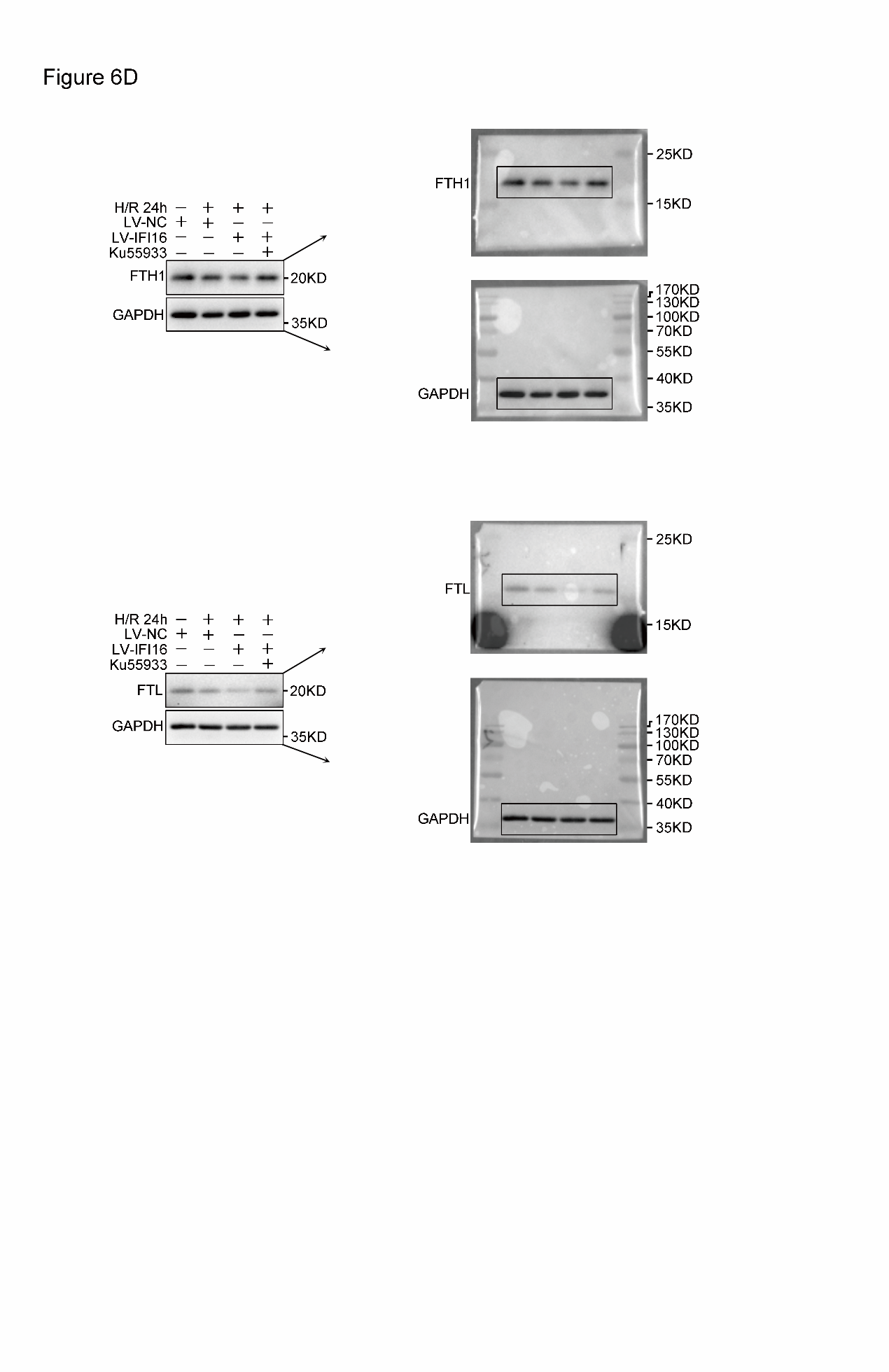


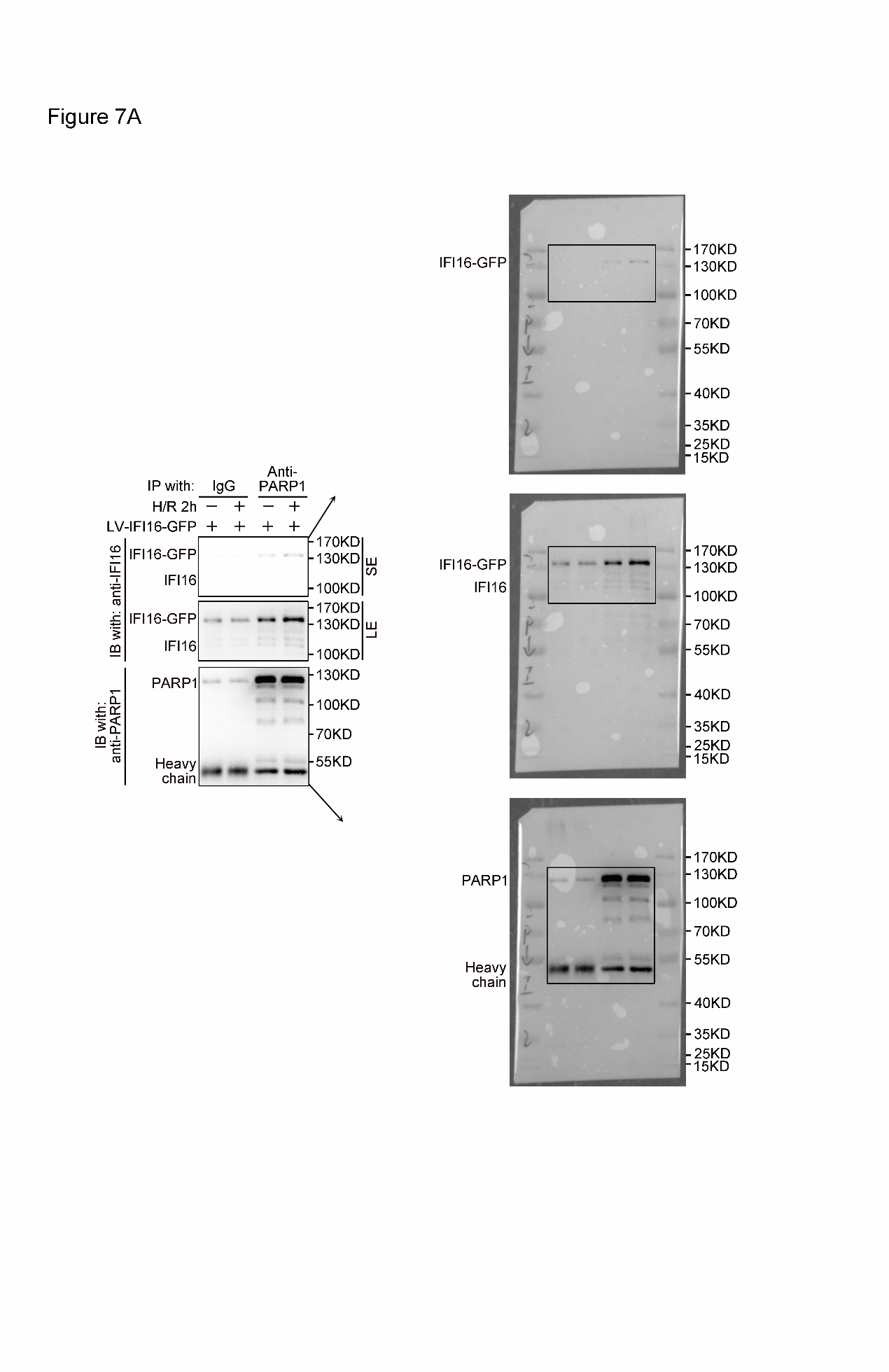


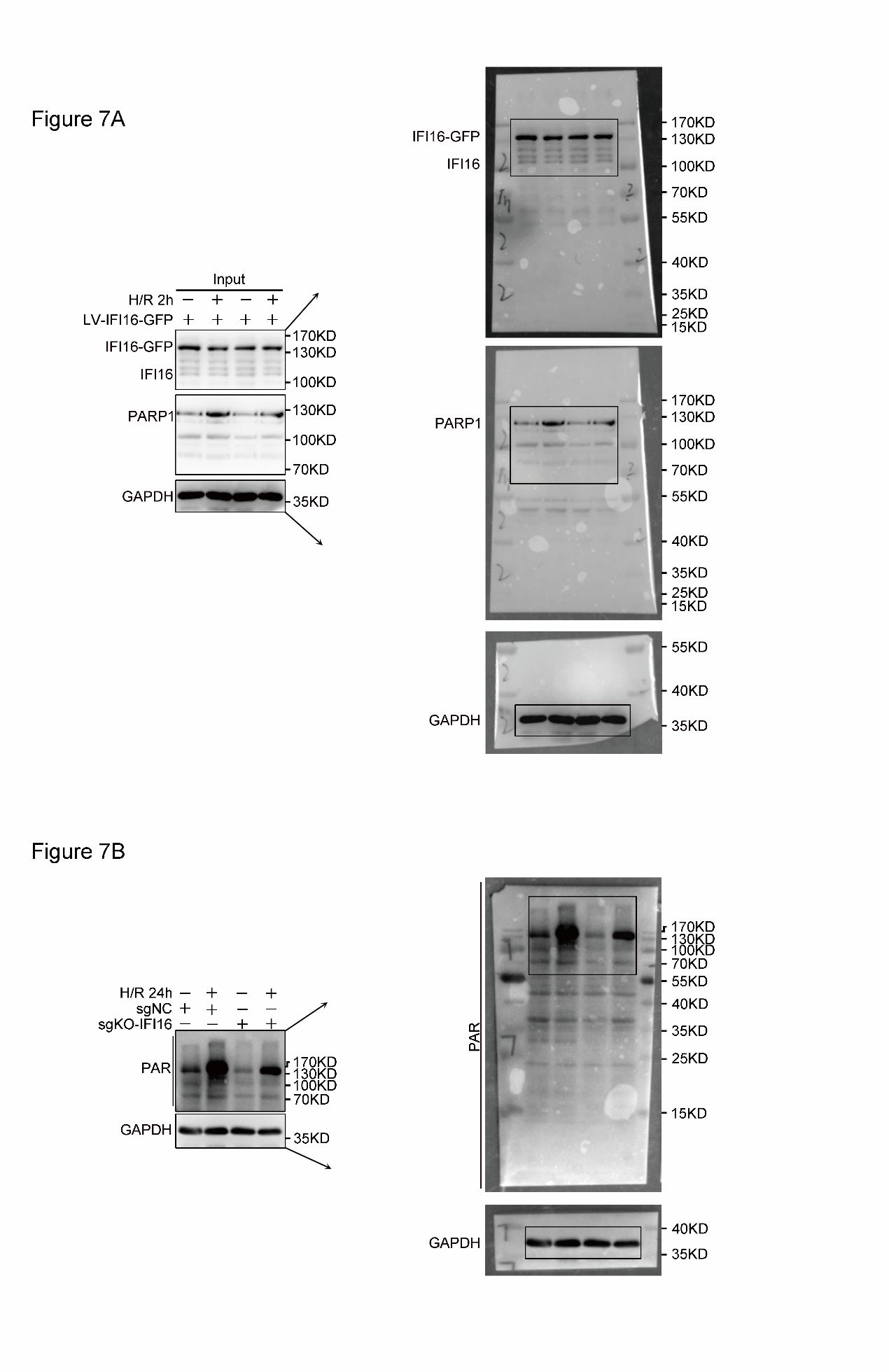


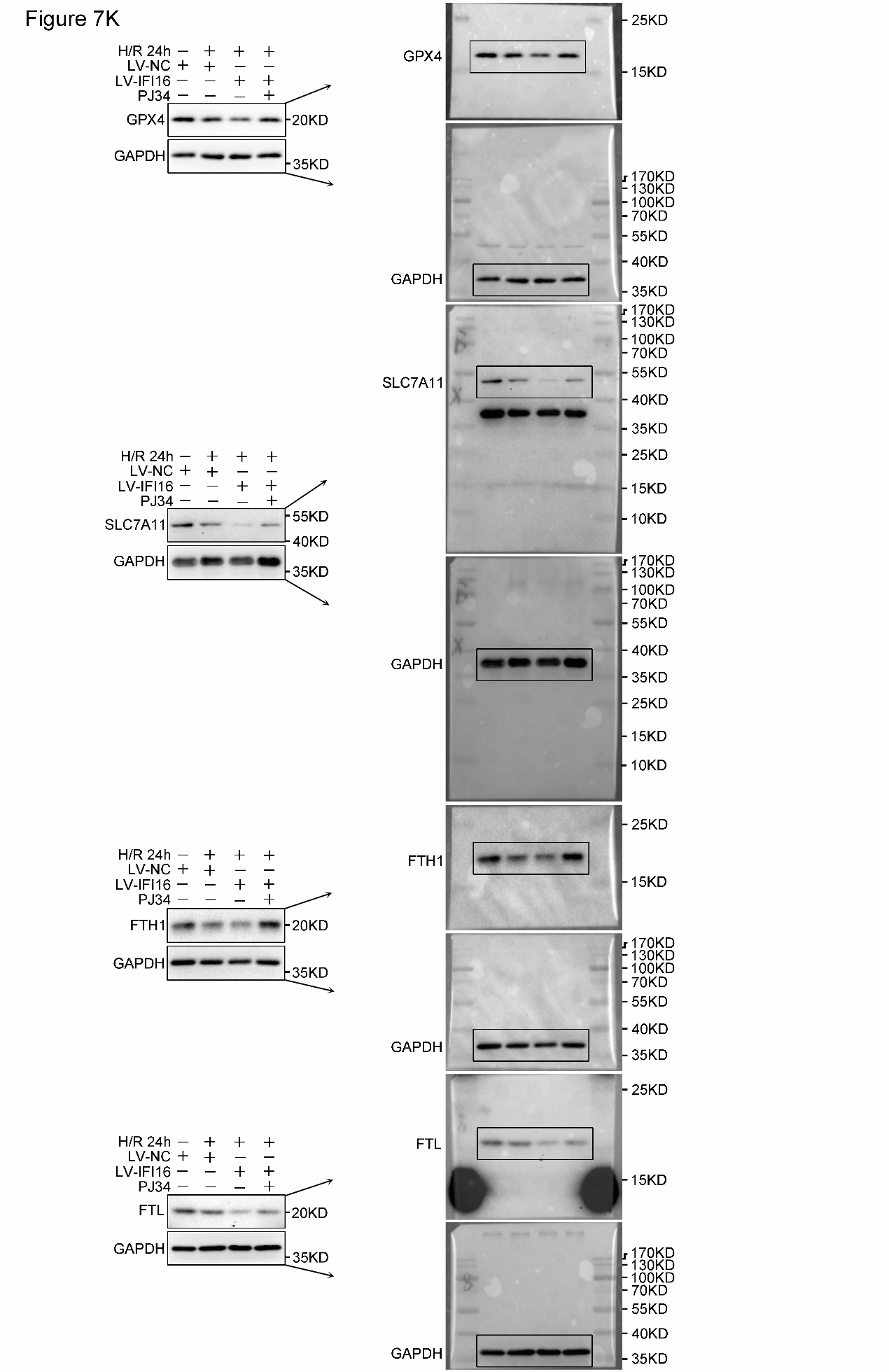


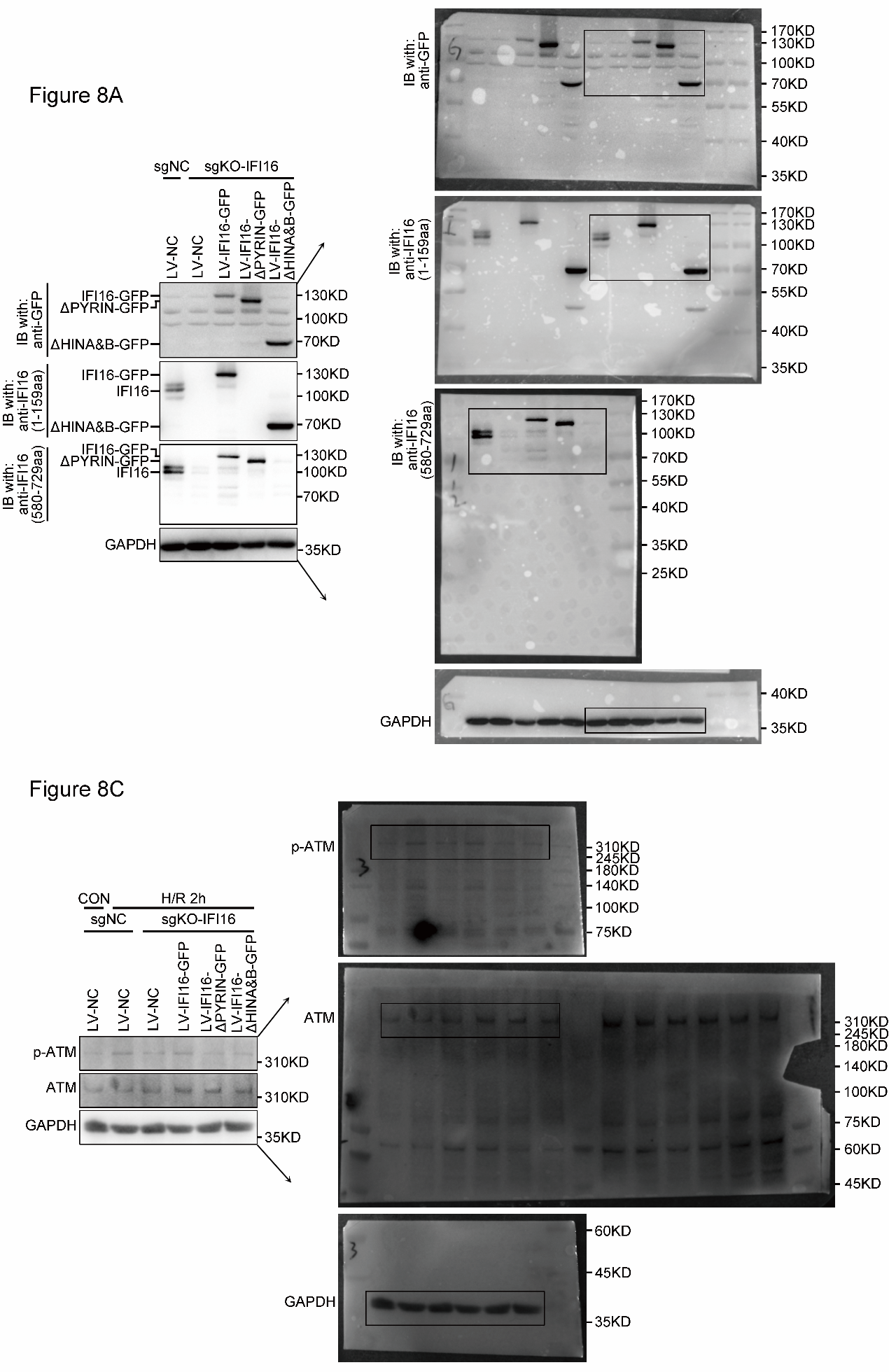


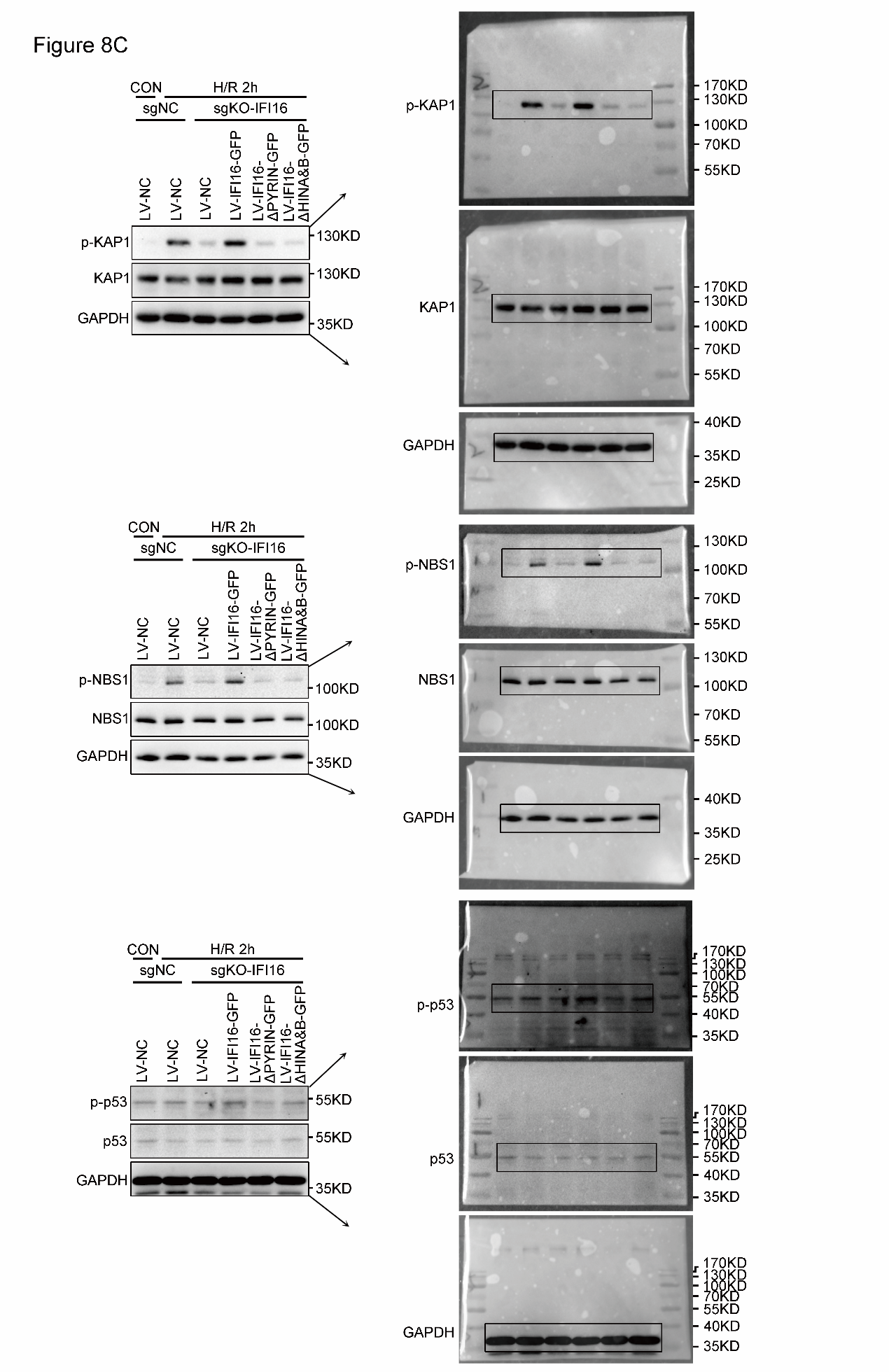


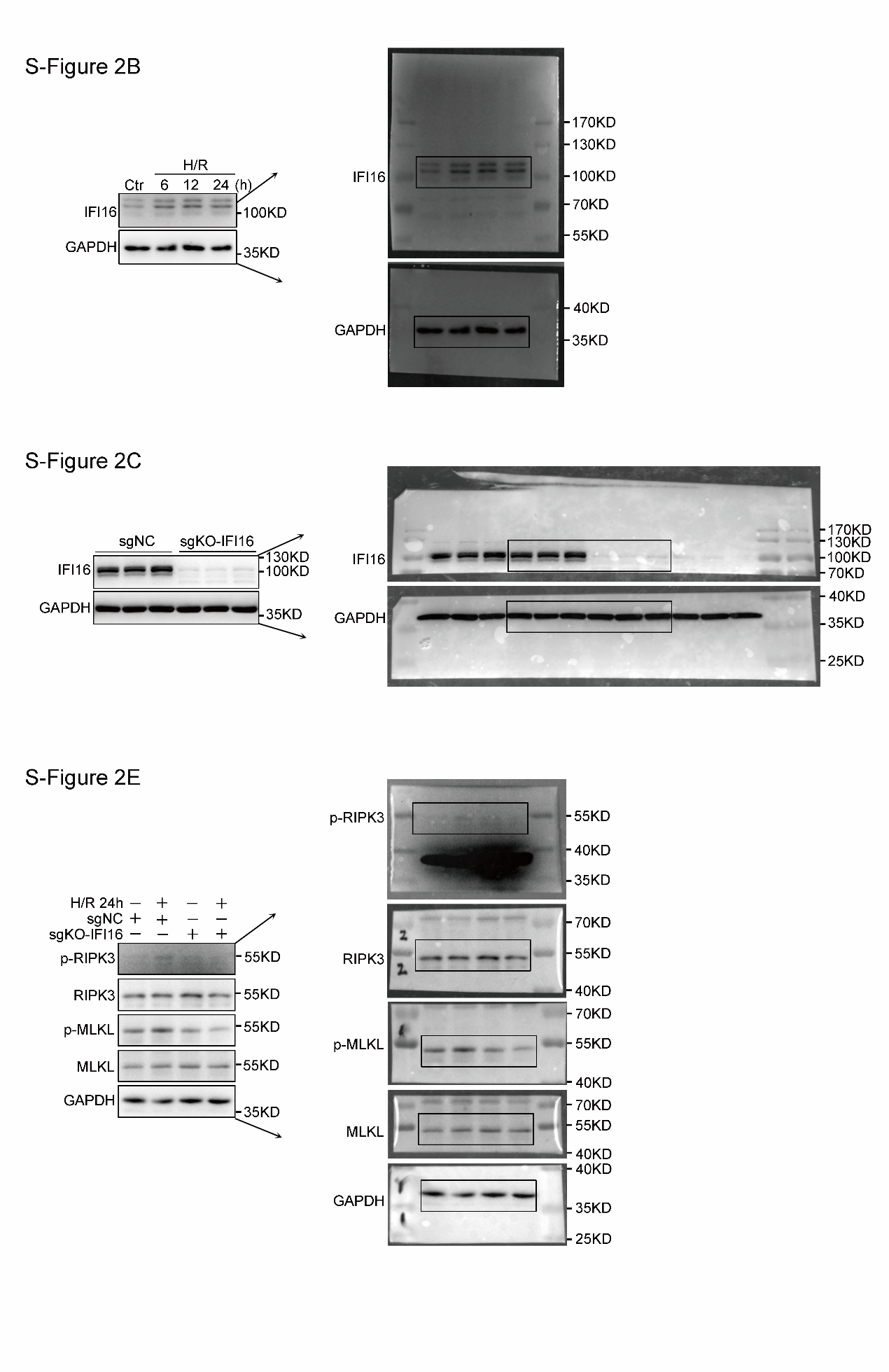


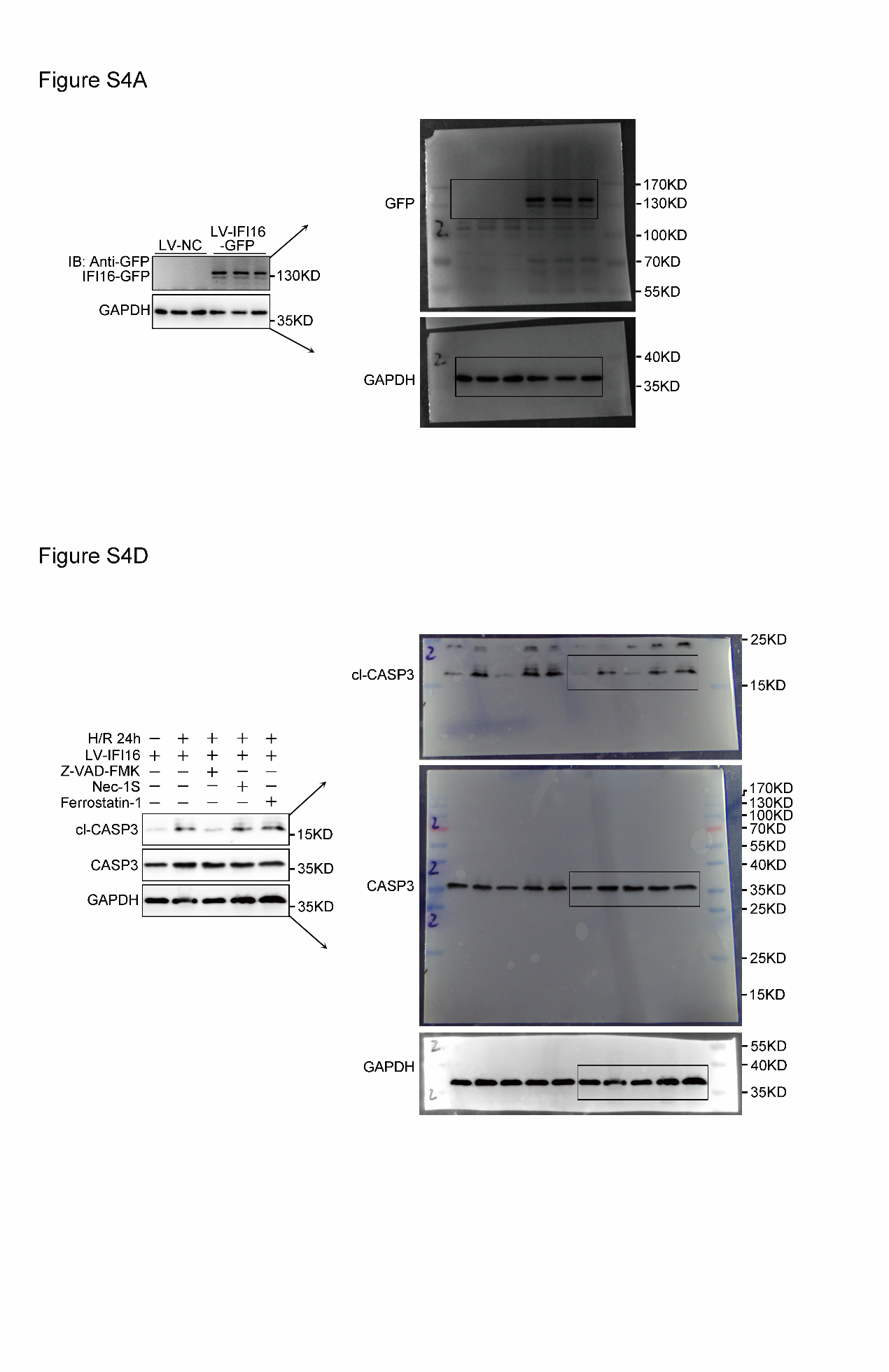


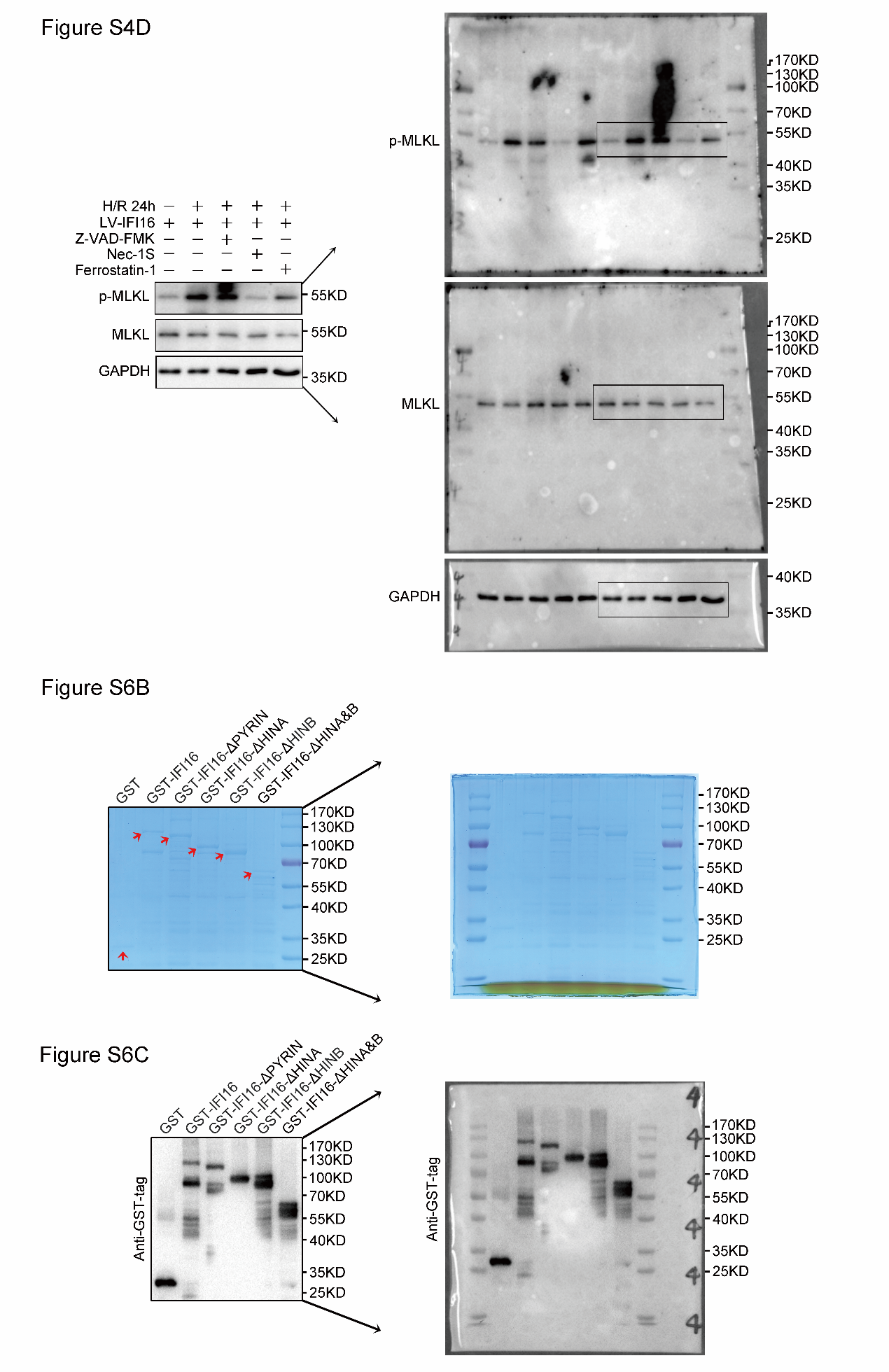


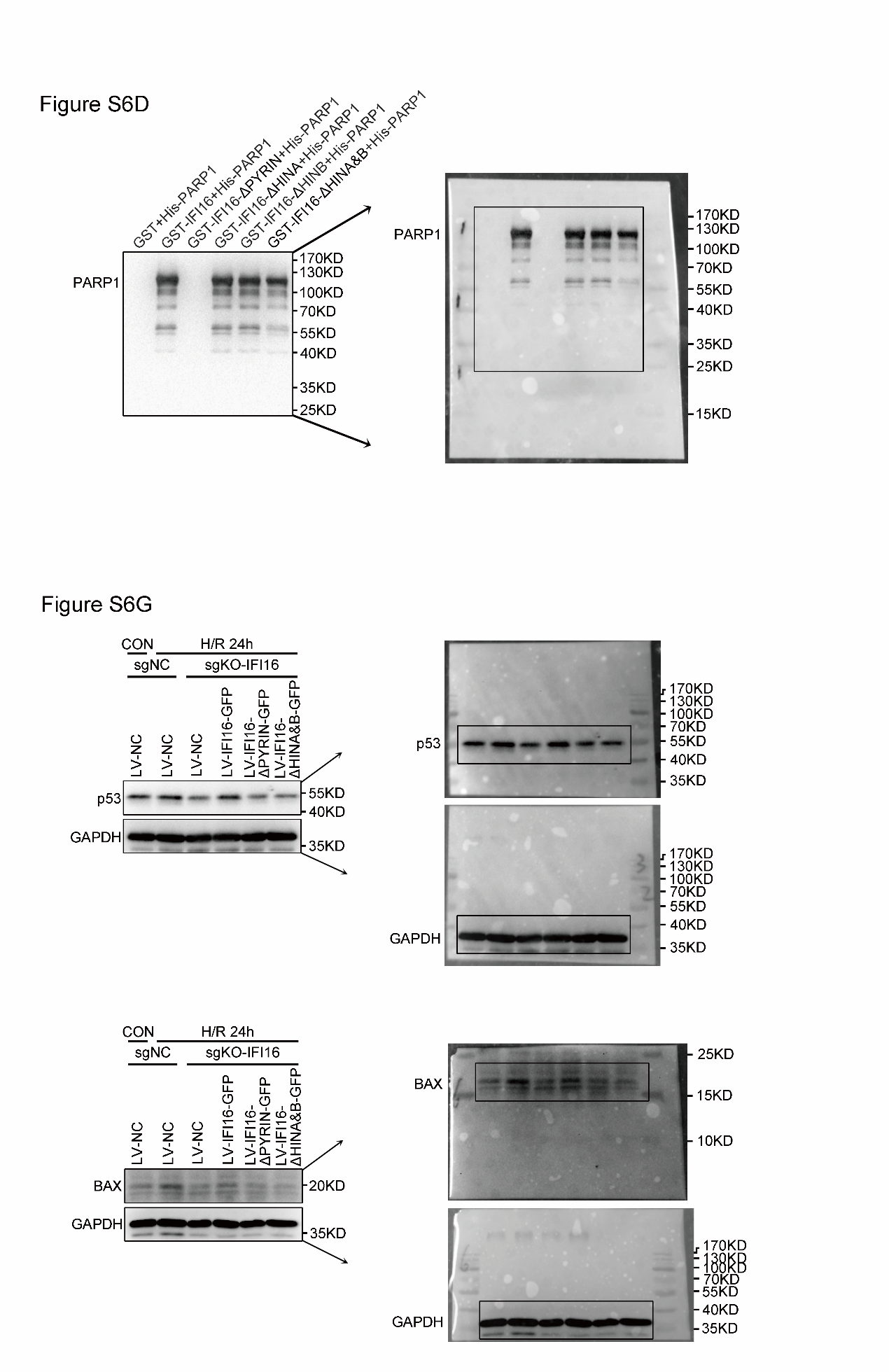


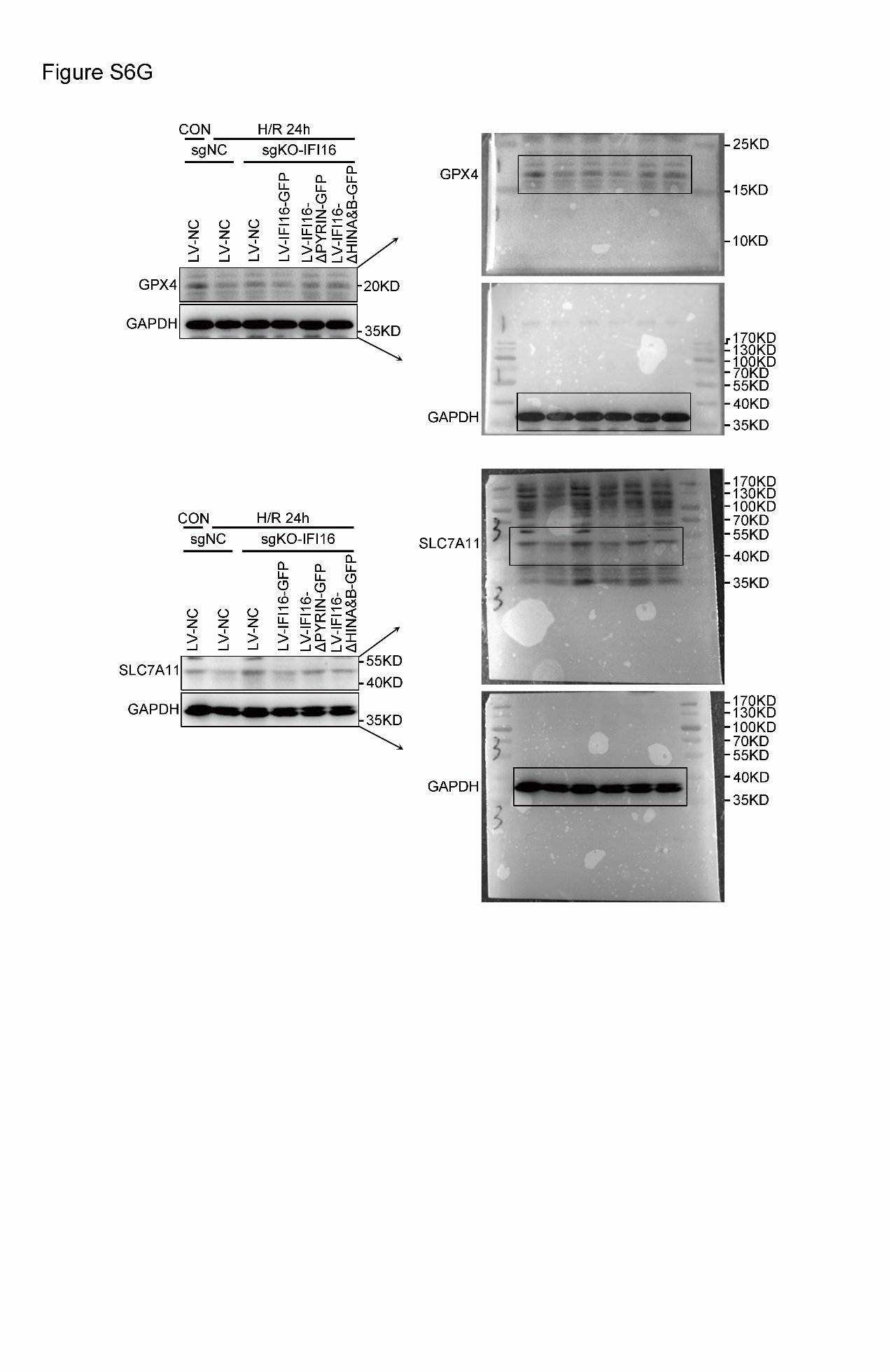

Supplement: Supplementary file 2 — The uncropped original images of electrophoretic blots and gels [file 41419_2026_8604_MOESM2_ESM.docx]
